# Retrieval-augmented reasoning reshapes collective reliability in text-based radiology question answering

**DOI:** 10.1016/j.patter.2026.101639

**Published:** 2026-08-19

**Authors:** Mina Farajiamiri, Jeta Sopa, Saba Afza, Lisa Adams, Felix Barajas Ordonez, Tri-Thien Nguyen, Mahshad Lotfinia, Sebastian Wind, Keno Bressem, Sven Nebelung, Daniel Truhn, Soroosh Tayebi Arasteh

**Affiliations:** 1 Lab for AI in Medicine, RWTH Aachen University, Aachen, Germany; 2 Pattern Recognition Lab, Friedrich-Alexander-Universität Erlangen-Nürnberg, Erlangen, Germany; 3 Department of Diagnostic and Interventional Radiology, TUM University Klinikum, School of Medicine and Health, Klinikum rechts der Isar, Technical University of Munich, Munich, Germany; 4 Department of Diagnostic and Interventional Radiology, University Hospital RWTH Aachen, Aachen, Germany; 5 Institute of Radiology, University Hospital Erlangen, Erlangen, Germany; 6 Erlangen National High Performance Computing Center, Friedrich-Alexander-Universität Erlangen-Nürnberg, Erlangen, Germany; 7 Department of Cardiovascular Radiology and Nuclear Medicine, TUM University Clinic, School of Medicine and Health, German Heart Center, Technical University of Munich, Munich, Germany; 8 Department of Urology, Stanford University, Stanford, CA, USA; 9 Department of Radiology, Stanford University, Stanford, CA, USA

**Keywords:** retrieval-augmented generation, large language models, question answering, radiology, collective reliability, reasoning

## Abstract

Retrieval-augmented reasoning pipelines are increasingly used to structure how large language models (LLMs) incorporate external evidence in clinical question answering, but their reliability under model variability remains unclear. We evaluated 34 LLMs on 169 expert-curated, text-only, multiple-choice radiology questions, comparing zero-shot inference with a fixed shared-evidence condition in which all models received identical structured evidence reports before answer selection. Primary endpoints were inter-model decision dispersion and panel-based robustness of correctness. Shared evidence reduced decision dispersion (median entropy 0.48→0.13; *p* = 5.6 × 10^−9^) and increased robustness of correctness (mean 0.74→0.81; *p* = 5.6 × 10^−9^). Majority consensus increased, and consensus strength remained strongly correlated with robustness. However, high agreement did not guarantee correctness, and rare high-consensus failures persisted. These findings show that shared evidence can improve reproducibility under model variability, but accuracy and agreement alone are insufficient to characterize reliability.

## Highlights

• Shared evidence reasoning sharply reduces inter-model disagreement in radiology QA
• Correct-answer reproducibility improves across 34 language models
• Evidence synthesis, not retrieval access alone, drives the robustness gains
• High agreement can still mask coordinated errors and rare robustness collapses

## The bigger picture

Large language models are being increasingly adopted in medicine, where they may soon contribute to answering important clinical questions and supporting diagnostic reasoning. But the model used in any such tool is not fixed: hospitals switch providers, update versions, and route questions across different systems to manage cost and availability. A tool that looks reliable on average can still be fragile if its answer depends on which model happens to respond. Most evaluations report only average accuracy and miss this hidden instability. We studied how a panel of 34 different language models behaves collectively on text-based radiology questions, comparing direct answering with a setup where every model receives the same retrieved, structured evidence before deciding. Sharing evidence pulled the models toward more consistent and more reproducibly correct answers, and we found that the synthesis of that evidence, not retrieval alone, drove the improvement. Yet the models sometimes agreed confidently on the same wrong answer. Reliable medical AI will therefore require evaluating not just whether a system is accurate, but whether it stays correct when the underlying model changes and whether agreement ever masks shared error.

## Graphical abstract

**Figure:**
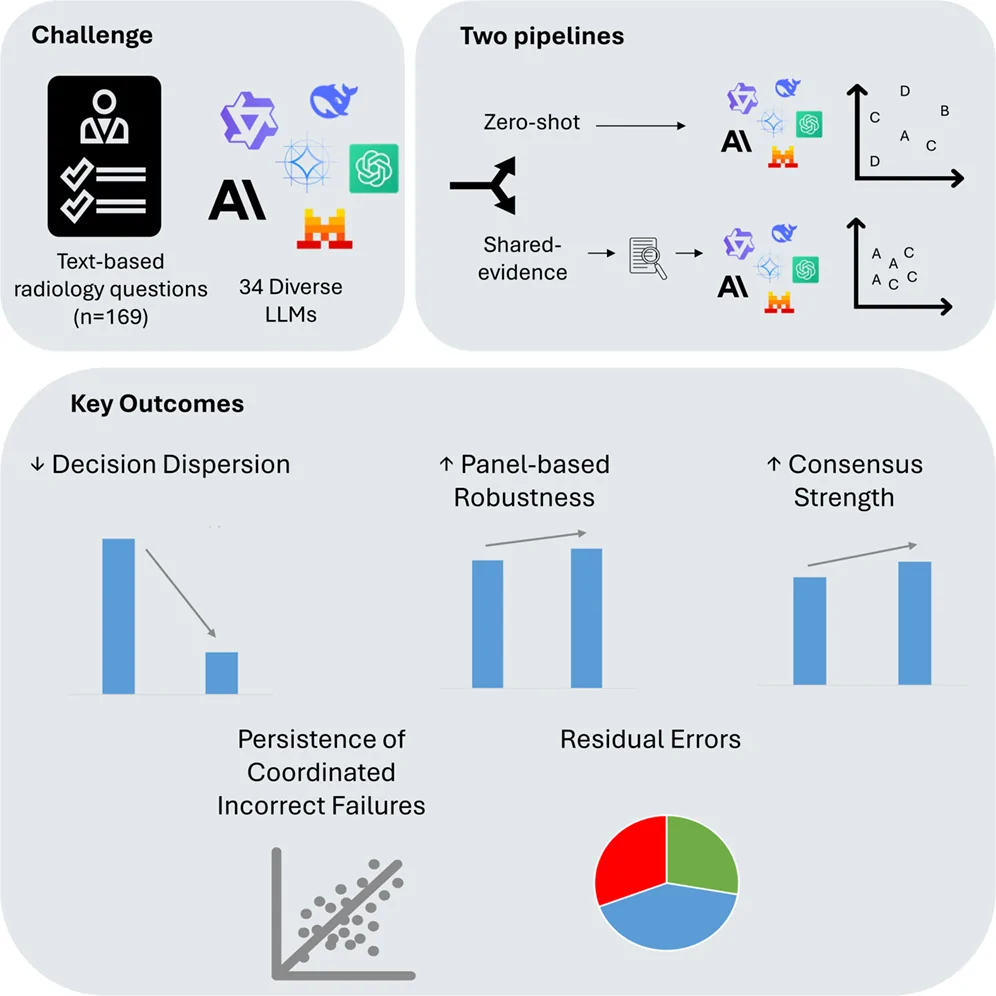

## Teaser

Can AI systems be trusted when the model behind them keeps changing? Farajiamiri et al. evaluate 34 language models on text-based radiology questions and show that giving every model the same structured evidence makes their answers more consistent and more reproducibly correct. The gains come from synthesizing evidence, not retrieval alone, yet models still sometimes agree on the same wrong answer support.

## Introduction

Large language models (LLMs) are increasingly incorporated into scientific, engineering, and medical question-answering workflows, where their outputs can shape expert reasoning and downstream decisions.^1^^,^^2^^,^^3^^,^^4^^,^^5^^,^^6^^,^^7^ In radiology, recent progress in retrieval-augmented and multi-step reasoning systems has improved performance on knowledge-intensive, text-based question answering (QA) tasks.^8^ However, improvements in mean accuracy alone are insufficient to characterize reliability when systems vary across model architectures, vendors, versions, and operational constraints.^9^^,^^10^ In practice, the deployed model is rarely fixed: organizations may switch providers, update model versions, or route queries across different backends to meet latency, cost, or availability constraints. Under such variability, a system can appear reliable in aggregate while remaining fragile if its answers depend strongly on model choice. This motivates model variability as a distinct reliability dimension. Inter-model variability is not merely noise to be averaged away; it can reveal sensitivity to context, instability of conclusions, and failure modes that are masked by reporting only mean performance.^11^^,^^12^

Retrieval-augmented reasoning can influence this variability in competing ways. Shared retrieval sources and structured evidence reports may align models toward similar conclusions, reducing decision dispersion and increasing apparent agreement.^13^^,^^14^ Conversely, the same shared structure can synchronize errors if retrieval is incomplete, if a synthesized report emphasizes misleading information, or if multiple models respond similarly to the same salient but incorrect cue.^15^^,^^16^ Such coordinated failures are important because apparent agreement may create false confidence even when the collective decision is wrong. It therefore remains unclear how shared-evidence retrieval and reasoning affect inter-model agreement, whether changes in agreement track correctness, whether correctness becomes more reproducible across heterogeneous models, and whether consensus remains informative once models are exposed to identical structured evidence.^11^^,^^17^^,^^18^ Related reliability signals also require careful interpretation. Users are often exposed to explanation length, structured rationales, or detailed reasoning traces, but these signals may not reliably indicate correctness.^19^^,^^20^ Similarly, the possible clinical consequence of residual errors cannot be inferred from correctness alone. An incorrect answer may be clinically minor or potentially consequential depending on the diagnostic context, downstream management, and opportunity for correction.^10^^,^^21^^,^^22^ However, severity assessment is itself subjective and context-dependent, so such analyses should be treated cautiously unless inter-rater reliability is strong. These considerations motivate a framework that separates decision concentration from correctness, consensus from robustness, and error frequency from potential clinical consequence.

To address these questions, we evaluate a controlled shared-evidence retrieval-augmented reasoning condition in text-based radiology QA (Figure 1). We compare zero-shot inference with a standardized condition in which a fixed orchestration pipeline retrieves information from a curated radiology knowledge source^23^ and synthesizes it into a structured evidence report.^8^^,^^13^^,^^24^ For a given question, all evaluated models receive the same report, and the final answer is always generated by the evaluated model itself. This design intentionally holds retrieval and evidence synthesis constant across models, allowing us to isolate how different models use identical structured evidence rather than comparing their independent retrieval, planning, or tool-use abilities. Accordingly, this study evaluates model behavior under one fixed shared-evidence design not retrieval-augmented reasoning systems in general.

**Figure 1:**
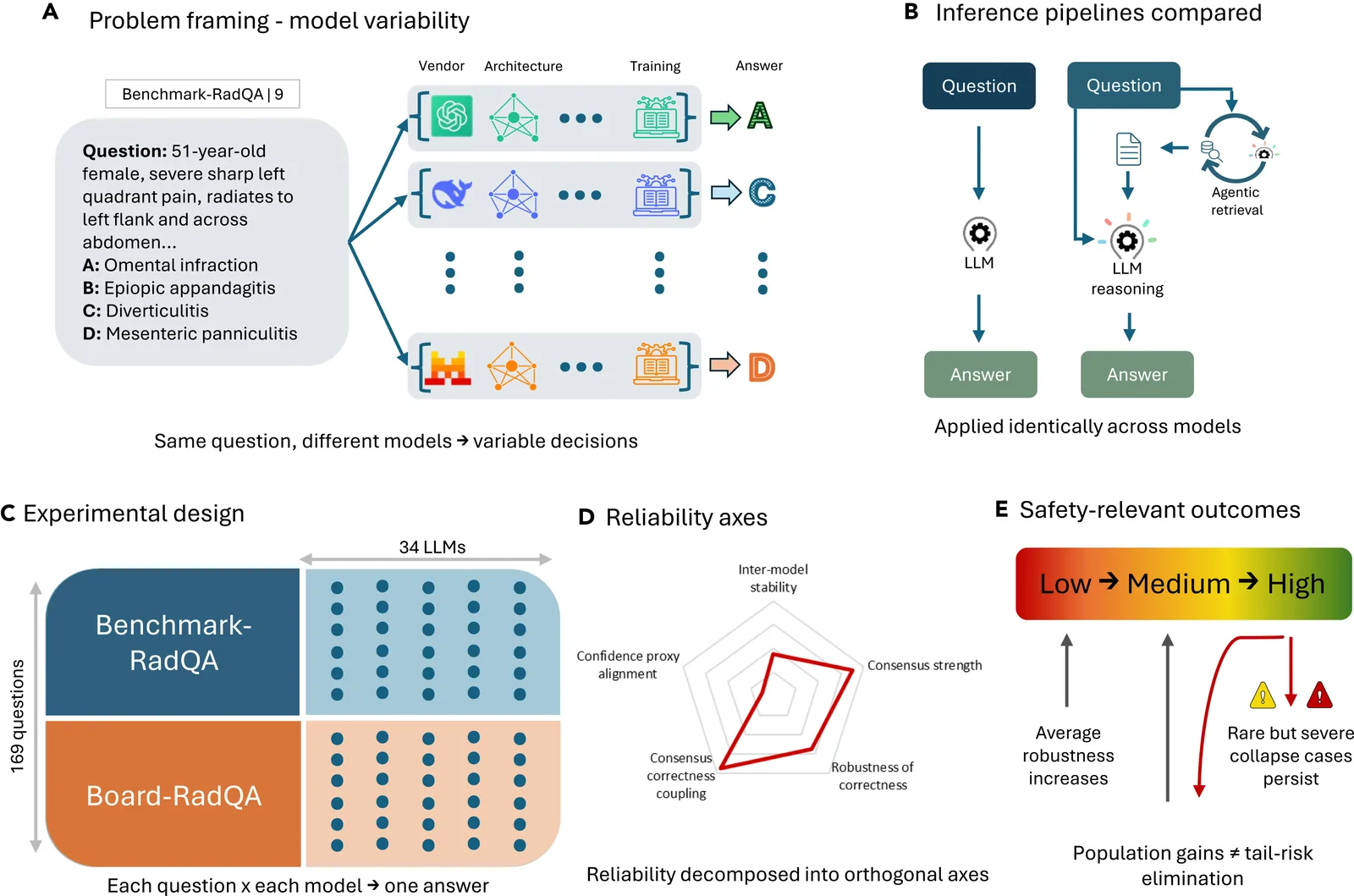
Study overview and experimental design (A) A single radiology multiple-choice question is presented to a heterogeneous panel of large language models (LLMs) that differ in vendor, architecture, and training. Although all models receive the same input, their final answers may diverge, illustrating model variability and motivating evaluation of collective reliability rather than single-model accuracy. (B) Zero-shot inference consists of direct question-to-answer generation by each model. In contrast, the shared-evidence retrieval-augmented pipeline incorporates structured multi-step reasoning and iterative evidence retrieval before answer selection. Both pipelines are applied identically across all 34 models to isolate the effect of inference strategy under controlled conditions. (C) A total of 169 multiple-choice radiology questions (Benchmark-RadQA, *n* = 104; Board-RadQA, *n* = 65) are answered independently by 34 LLMs under both zero-shot and shared-evidence conditions. Each question-model pair yields one discrete answer, enabling paired per-question comparisons across inference strategies. (D) Collective behavior is decomposed into orthogonal metrics: inter-model stability (entropy), consensus strength (majority fraction), robustness of correctness (fraction of models correct), coupling between consensus and correctness, and alignment between verbosity and correctness as a confidence proxy. This multidimensional framework separates coordination structure from validity. (E) Panel-based robustness scores are stratified into low, medium, and high bins to characterize reliability regimes. Shared-evidence reasoning is evaluated for both population-level robustness gains and the persistence of rare but severe collapse cases, emphasizing that improvements in average robustness do not necessarily eliminate tail-risk failure modes.

We apply this framework to 34 LLMs evaluated on 169 expert-curated, text-only, multiple-choice radiology questions from Benchmark-RadQA (*n* = 104), sourced from the RadioRAG study,^24^ and Board-RadQA (*n* = 65), sourced from the RaR study.^8^ The primary endpoints are inter-model decision stability, quantified by answer-distribution entropy,^18^^,^^25^ and panel-based robustness of correctness, defined as the fraction of models in the evaluated 34-model panel that answer a question correctly.^26^ Secondary and exploratory analyses examine majority-consensus behavior, coupling between consensus strength and robustness,^18^^,^^27^ rare high-consensus low-robustness failures,^11^^,^^15^ verbosity-correctness associations,^19^^,^^20^ and clinician-assigned severity labels for incorrect answer options.^10^^,^^21^^,^^22^^,^^28^ By separating decision concentration from correctness, robustness from consensus, and error frequency from potential consequence, this study provides a controlled framework for assessing reliability under model variability while remaining limited to text-based radiology QA rather than image interpretation, multimodal clinical workflows, or prospective clinical deployment.

## Results

We evaluated zero-shot inference and shared-evidence retrieval-augmented reasoning across 169 multiple-choice radiology questions drawn from the Benchmark-RadQA (*n* = 104) and Board-RadQA (*n* = 65) datasets (Table S1), answered by 34 LLMs (model specifications in Table S2). The primary data comprised per-question discrete answer choices from each model under each inference condition, enabling paired, question-level comparisons. Pooled paired analyses across all 169 questions constitute the primary confirmatory comparisons, while dataset-stratified results are reported in Note S1 to assess directional consistency across subsets. In accordance with the prespecified outcome hierarchy, inter-model decision stability and panel-based robustness of correctness were treated as the primary endpoints. Consensus behavior, consensus-robustness coupling, verbosity analyses, and clinical severity annotations were treated as secondary or exploratory analyses. These secondary analyses are reported to characterize collective behavior and possible safety-relevant patterns, but they are not interpreted as independently confirmatory endpoints. For descriptive context, Table 1 reports single-model accuracy under zero-shot and shared-evidence inference for each of the 34 LLMs.

**Table 1:** Accuracy of language models across zero-shot prompting and the shared-evidence model over all 169 questions

Model name	Zero-shot		Shared-evidence		*p* value
	Accuracy (%)	Total correct (*n*)	Accuracy (%)	Total correct (*n*)	
Claude-Sonnet-4.6	85 ± 3 [80, 90]	144	88 ± 3 [82, 92]	148	0.577
Gemini-3.1-Pro	82 ± 3 [76, 87]	138	91 ± 2 [86, 95]	154	0.012
MiniMax-M2.5	79 ± 3 [73, 86]	134	80 ± 3 [74, 86]	135	>0.999
GLM-5	82 ± 3 [76, 88]	139	85 ± 3 [80, 91]	144	0.527
LFM2.5-1.2B-Thinking	56 ± 4 [49, 63]	94	76 ± 3 [69, 82]	129	<0.001
Kimi-K2.5	83 ± 3 [78, 89]	141	85 ± 3 [79, 91]	144	0.729
Palmyra-X5	86 ± 3 [81, 91]	146	86 ± 3 [80, 91]	145	>0.999
MiMo-V2-Flash	79 ± 3 [72, 85]	133	87 ± 3 [82, 92]	147	0.063
Llama4-Scout-16E	82 ± 3 [76, 88]	139	85 ± 3 [79, 90]	143	0.585
Llama3.3-8B	69 ± 4 [63, 76]	117	75 ± 3 [67, 80]	126	0.275
Llama3.3-70B	80 ± 3 [74, 86]	135	86 ± 3 [80, 91]	145	0.144
Llama3-Med42-8B	63 ± 4 [56, 70]	107	74 ± 4 [67, 80]	125	0.024
Llama3-Med42-70B	75 ± 3 [69, 82]	127	79 ± 3 [73, 85]	134	0.417
DeepSeek R1-70B	84 ± 3 [79, 89]	142	84 ± 3 [78, 89]	142	>0.999
DeepSeek-R1	88 ± 2 [82, 92]	148	84 ± 3 [78, 89]	142	0.343
DeepSeek-V3	84 ± 3 [79, 89]	142	89 ± 3 [83, 93]	150	0.218
GPT-5	84 ± 3 [78, 89]	142	87 ± 3 [82, 92]	147	0.402
GPT-5.2	79 ± 3 [73, 85]	134	87 ± 3 [82, 92]	147	0.035
o3	85 ± 3 [79, 90]	144	89 ± 2 [84, 93]	150	0.239
GPT-3.5-turbo	64 ± 4 [57, 71]	108	77 ± 3 [70, 83]	130	0.012
GPT-4-turbo	77 ± 3 [70, 83]	130	82 ± 3 [76, 88]	138	0.277
Mistral Large (123B)	81 ± 3 [75, 87]	137	86 ± 3 [80, 91]	146	0.166
Ministral-8B	54 ± 4 [46, 62]	91	76 ± 3 [69, 82]	128	<0.001
MedGemma-4B-it	64 ± 4 [57, 71]	108	75 ± 3 [68, 81]	127	0.024
MedGemma-27B-text-it	79 ± 3 [72, 85]	133	84 ± 3 [78, 89]	142	0.220
Gemma-3-4B-it	51 ± 4 [44, 59]	86	71 ± 3 [64, 78]	121	<0.001
Gemma-3-27B-it	71 ± 3 [64, 78]	120	83 ± 3 [78, 89]	141	0.009
Qwen3-8B	75 ± 3 [68, 81]	127	81 ± 3 [75, 86]	137	0.218
Qwen3-235B	86 ± 3 [81, 91]	146	85 ± 3 [79, 91]	144	0.877
Qwen2.5-0.5B	37 ± 4 [30, 45]	63	48 ± 4 [41, 56]	81	0.079
Qwen2.5-3B	63 ± 4 [57, 71]	107	73 ± 3 [66, 80]	124	0.038
Qwen2.5-7B	63 ± 4 [57, 70]	107	78 ± 3 [71, 84]	132	0.002
Qwen2.5-14B	74 ± 3 [68, 81]	126	79 ± 3 [73, 85]	134	0.354
Qwen2.5-70B	79 ± 3 [73, 85]	134	84 ± 3 [78, 89]	142	0.229

Accuracy is reported in percentage as mean ± standard deviation, with 95% confidence intervals shown in brackets. Results are based on 169 questions, using bootstrapping with 1,000 repetitions and replacement while preserving pairing. *p* values were calculated for each model using McNemar’s test on paired outcomes between the zero-shot and shared-evidence methods and adjusted for multiple comparisons using the false discovery rate. A *p* value <0.05 was considered statistically significant. Accuracy is presented alongside total correct answers per method.

### Shared-evidence reasoning alters inter-model decision stability

We first quantified how shared-evidence retrieval-augmented reasoning^8^ changes inter-model decision stability, operationalized as the Shannon entropy^25^ of the answer distribution across the model panel for each question. Across all 169 questions, shared-evidence reasoning yielded lower entropy than zero-shot inference (Table 2; Figure 2), indicating that model decisions were more concentrated under the shared-evidence pipeline. The median entropy decreased from 0.48 (interquartile range [IQR] 0.55) under zero-shot to 0.13 (IQR 0.51) under shared-evidence reasoning, and the mean decreased from 0.50 to 0.40. The paired per-question shift was significant (*p* = 5.6 × 10^−9^); rank-biserial r = −0.93), with a median ΔH of −0.13 and mean ΔH of −0.19, demonstrating an overall reduction in dispersion across models. Per-question comparisons showed that the effect was not uniform. In the pooled dataset, entropy decreased in 115 of 169 questions (68%), increased in 31 (18%), and remained unchanged in 23 (14%). Table S3 provides representative per-question examples illustrating both stabilizing cases (negative ΔH) and occasional destabilizations (positive ΔH). Importantly, entropy captures coordination rather than correctness: lower entropy reflects stronger alignment among models, not necessarily higher validity. This distinction motivates subsequent analyses examining whether increased stability translates into more robust correctness or more reliable consensus behavior under model variability.

**Table 2:** Inter-model decision stability under zero-shot vs. shared-evidence inference

Metric	Pooled datasets	Board-RadQA dataset	Benchmark-RadQA dataset
Questions [n]	169	65	104
Entropy (zero-shot), median (IQR)	0.48 (0.55)	0.35 (0.50)	0.53 (0.56)
Entropy (shared-evidence), median (IQR)	0.13 (0.51)	0.13 (0.13)	0.24 (0.57)
Entropy (zero-shot), mean	0.50	0.41	0.55
Entropy (shared-evidence), mean	0.40	0.20	0.38
Paired entropy change (ΔH), median	−0.13	−0.13	−0.13
Paired entropy change (ΔH), mean	−0.19	−0.20	−0.18
Questions with ΔH < 0/> 0/= 0, n (%)	115 (68%)/31 (18%)/23 (14%)	46 (71%)/8 (12%)/11 (17%)	69 (66%)/23 (22%)/12 (12%)
*p* value	5.6 × 10^−9^	9.6 × 10^−6^	9.4 × 10^−9^
Rank-biserial correlation (r)	−0.93	−0.94	−0.64

Inter-model decision stability was quantified for each question using Shannon entropy of the answer distribution across the 34-model panel, with lower entropy indicating stronger agreement among models. Entropy values are reported for zero-shot and shared-evidence retrieval-augmented inference across pooled questions and dataset-specific subsets. Paired changes in decision stability (ΔH) were computed on a per-question basis as shared-evidence minus zero-shot entropy; negative ΔH values indicate increased stability under shared-evidence inference, whereas positive values indicate decreased stability. Summary statistics are reported as medians with interquartile ranges and as means. The distribution of per-question entropy changes is additionally summarized by the number and proportion of questions exhibiting decreased (ΔH < 0), increased (ΔH > 0), or unchanged (ΔH = 0) entropy. Statistical significance of paired entropy changes was assessed using a two-sided Wilcoxon signed-rank test on paired per-question entropy values, with rank-biserial correlation (r) reported as an effect size for the paired comparison.

**Figure 2:**
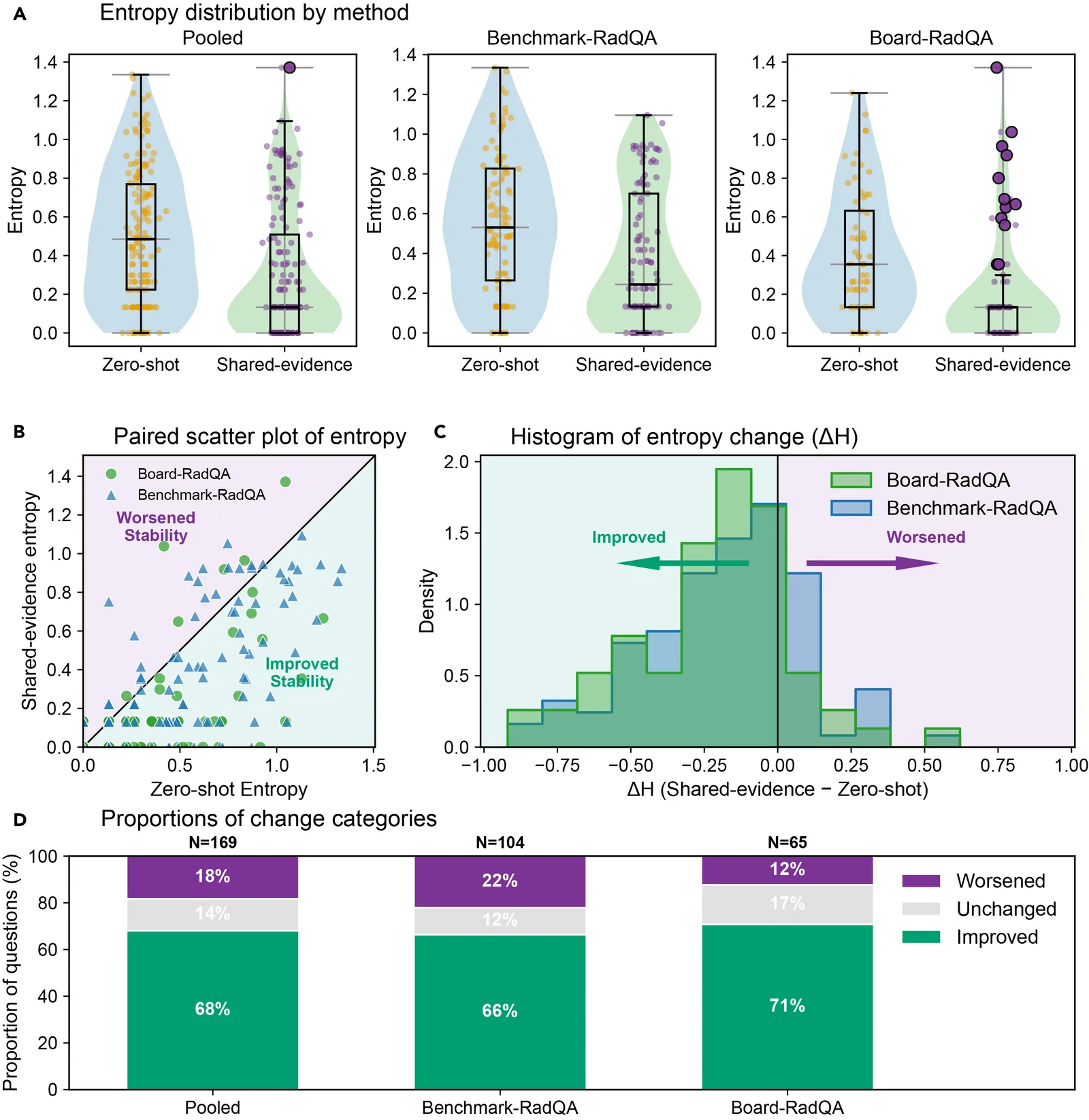
Inter-model decision stability under zero-shot vs. shared-evidence retrieval-augmented inference Inter-model stability was quantified for each question using Shannon entropy of the answer distribution across the 34-model panel, with lower entropy indicating stronger concentration of decisions. Results are shown for pooled questions (*n* = 169) and separately for Benchmark-RadQA (*n* = 104) and Board-RadQA (*n* = 65). (A) Violin plots with embedded boxplots display entropy distributions under zero-shot and shared-evidence inference for pooled and dataset-specific subsets. Points represent per-question entropy values; boxplots show median and interquartile range. (B) Paired scatterplot of per-question entropy values under zero-shot (*x* axis) vs. shared-evidence inference (*y* axis). The identity line indicates no change; points below the line reflect reduced entropy (improved stability) under shared-evidence inference, whereas points above indicate increased entropy. (C) Histograms of per-question entropy change (ΔH = shared-evidence − zero-shot) for each dataset. Negative ΔH values correspond to increased stability, and positive values indicate decreased stability. (D) Proportions of questions exhibiting improved (ΔH < 0), unchanged (ΔH = 0), or worsened (ΔH > 0) stability in pooled and dataset-specific analyses. Statistical significance of paired entropy changes was assessed using two-sided Wilcoxon signed-rank tests on per-question entropy values.

To address potential concerns related to the multiple-choice conversion of the RadioRAG-derived Benchmark-RadQA items, we repeated the primary entropy analysis in the non-converted Board-RadQA subset alone. In Board-RadQA (*n* = 65), shared-evidence inference again reduced inter-model entropy, with median entropy decreasing from 0.35 under zero-shot inference to 0.13 under shared-evidence inference (paired median ΔH = −0.13; *p* = 9.6 × 10^−6^, r = −0.94). Comparable reductions were observed in the Benchmark-RadQA subset (*n* = 104), where median entropy decreased from 0.53 to 0.24 (paired median ΔH = −0.13; *p* = 9.4 × 10^−9^, r = −0.64). These findings indicate that the primary collective-behavior result, reduced inter-model disagreement under shared-evidence inference, was directionally consistent in the non-converted subset and was therefore not attributable solely to multiple-choice conversion (Note S2).

### Structured evidence synthesis, not retrieval access alone, drives the gains

To identify which component of the shared-evidence pipeline drives the observed effects, we compared three conditions on a matched 15-model subset: zero-shot inference, standard retrieval-augmented inference (standard RAG), and shared-evidence retrieval-augmented reasoning (Note S3). The three metrics shifted monotonically across conditions. Median inter-model entropy decreased from 0.70 under zero-shot to 0.57 under standard RAG and to 0.35 under shared-evidence reasoning; median majority fraction increased from 0.87 to 0.87 to 0.93; and median panel-based robustness increased from 0.80 to 0.87 to 0.93. Introducing retrieval access alone (standard RAG vs. zero-shot) significantly reduced entropy (mean ΔH = −0.11; *p* = 0.003; r = −0.29) and increased majority fraction (mean ΔM = +0.04; *p* = 0.009; r = 0.27) but did not significantly change panel-based robustness (mean ΔR = +0.02; *p* = 0.092). Adding structured evidence synthesis on top of retrieval (shared-evidence vs. standard RAG) produced further significant gains across all three primary and secondary metrics: entropy decreased (mean ΔH = −0.10; *p* = 0.011; r = −0.25), majority fraction increased (mean ΔM = +0.03; *p* = 0.003; r = 0.30), and panel-based robustness increased (mean ΔR = +0.03; *p* = 0.003; r = 0.22). The full contrast (shared-evidence vs. zero-shot) was significant for all three metrics, with the largest effect sizes (entropy *p* = 6.6 × 10^−7^, r = −0.50; majority fraction *p* = 6.7 × 10^−7^, r = 0.51; robustness *p* = 2.9 × 10^−4^, r = 0.38). These results indicate that retrieval access alone concentrates model decisions but is not sufficient to improve cross-model reproducibility of correctness, whereas the structured evidence synthesis step contributes additional, significant improvements in both decision concentration and panel-based robustness beyond standard RAG.

### Changes in consensus strength do not reliably track correctness

We next performed a secondary analysis to test whether shared-evidence retrieval-augmented reasoning changes the strength of inter-model consensus and whether shifts in consensus preferentially align with correct decisions. For each question and method, we computed the majority fraction, defined as the proportion of models selecting the modal answer option, and evaluated whether the resulting majority decision matched the reference standard (Table 3; Table S4; Figure 3). Across all 169 questions, shared-evidence reasoning increased consensus strength. The median majority fraction increased from 0.85 (IQR 0.21) under zero-shot inference to 0.97 (IQR 0.18) under shared-evidence reasoning. The paired per-question comparison, restricted to non-zero ΔM pairs, showed a significant shift (*p* = 2.9 × 10^−5^), with a positive median ΔM of 0.03, indicating that agreement typically increased under the shared-evidence pipeline. Per-question categorization showed that agreement increased with a correct majority in 95 of 169 questions (56%), increased with an incorrect majority in 11 (7%), decreased in 30 (18%), and remained unchanged in 33 (20%). Thus, agreement amplification occurred more often than agreement loss and was more frequently associated with correct majorities, but it was not exclusively correctness-favorable. In a measurable subset of cases, shared-evidence reasoning concentrated models around an incorrect answer. These findings indicate that shared-evidence retrieval strengthens inter-model consensus at the population level, yet consensus strength remains an imperfect indicator of decision validity.

**Table 3:** Majority-vote agreement strength and correctness under zero-shot vs. shared-evidence reasoning

Metric	Pooled dataset	Board-RadQA dataset	Benchmark-RadQA dataset
Questions [n]	169	65	104
Median majority fraction (zero-shot)	0.85	0.91	0.82
Median majority fraction (shared-evidence)	0.97	0.97	0.94
Median ΔM (shared-evidence − zero-shot)	0.03	0.03	0.06
IQR majority fraction (zero-shot)	0.21	0.18	0.27
IQR majority fraction (shared-evidence)	0.18	0.06	0.21
Questions with agreement ↑ and majority correct	95 (56%)	40 (62%)	55 (53%)
Questions with agreement ↑ and majority incorrect	11 (7%)	1 (2%)	10 (10%)
Questions with agreement ↓	30 (18%)	10 (15%)	20 (19%)
Questions with no change in agreement	33 (20%)	14 (22%)	19 (18%)
Non-zero ΔM pairs	136	51	85
*p* value	2.9 × 10^−5^	1.5 × 10^−4^	5.7 × 10^−7^

Inter-model agreement strength was quantified for each question as the majority fraction, defined as the proportion of models selecting the modal answer option among the 34-model panel. Values are reported for zero-shot and shared-evidence retrieval-augmented inference across pooled questions and dataset-specific subsets. Paired changes in agreement strength (ΔM) were computed on a per-question basis as shared-evidence minus zero-shot majority fraction. Positive ΔM indicates increased agreement under shared-evidence reasoning, while negative ΔM indicates decreased agreement. Questions were categorized according to whether agreement increased with a correct majority, increased with an incorrect majority, decreased, or remained unchanged. Statistical significance of paired agreement changes was assessed using a two-sided Wilcoxon signed-rank test applied to non-zero ΔM pairs only. All reported *p* values correspond to this paired non-parametric test.

**Figure 3:**
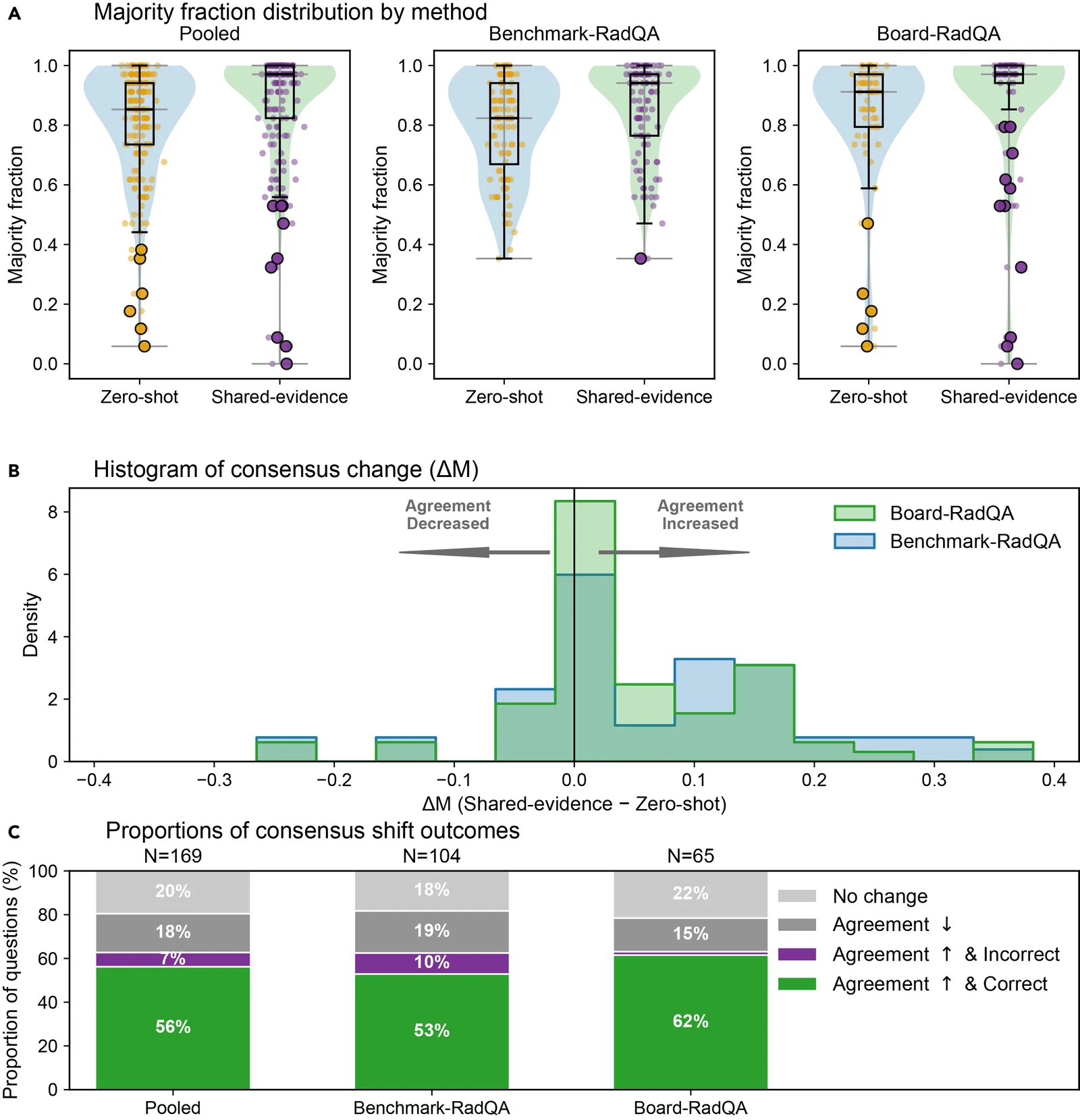
Inter-model consensus strength and correctness under zero-shot vs. shared-evidence inference Inter-model agreement was quantified for each question as the majority fraction (M), defined as the proportion of the 34 models selecting the modal answer option. Results are shown for pooled questions (*n* = 169) and separately for Benchmark-RadQA (*n* = 104) and Board-RadQA (*n* = 65). (A) Violin plots with embedded boxplots display the distribution of majority fractions under zero-shot and shared-evidence retrieval-augmented inference. Points represent per-question values; boxplots indicate median and interquartile range. (B) Histograms of paired changes in agreement strength (ΔM = shared-evidence − zero-shot). Positive ΔM values indicate increased consensus under shared-evidence inference, whereas negative values indicate decreased consensus. Distributions are shown separately for each dataset. (C) Proportions of questions categorized by consensus shift outcome: agreement increased with correct majority, agreement increased with incorrect majority, agreement decreased, or no change. Statistical significance of paired agreement changes was assessed using a two-sided Wilcoxon signed-rank test on non-zero ΔM pairs.

As a secondary analysis, we also examined majority fraction across subsets. Shared-evidence inference increased the median majority fraction from 0.91 to 0.97 in Board-RadQA (median ΔM = 0.03; *p* = 1.5 × 10^−4^) and from 0.82 to 0.94 in Benchmark-RadQA (median ΔM = 0.06; *p* = 5.7 × 10^−7^). In the pooled analysis, the median majority fraction increased from 0.85 to 0.97 (median ΔM = 0.03; *p* = 2.9 × 10^−5^). These results support that shared-evidence inference strengthened panel-level convergence across subsets, although this secondary metric was not interpreted as an independent marker of correctness (Note S2).

It should be noted that, for this analysis, Benchmark-RadQA includes 104 questions with four answer options, whereas Board-RadQA includes 65 questions with five answer options. We therefore decided to conduct the analysis using the four answer options common to both datasets. We considered that adding a non-existent fifth option to Benchmark-RadQA solely for consistency would not be appropriate and could introduce misleading values. Moreover, in Board-RadQA, the fifth option is the correct answer in only three questions; therefore, excluding this option is expected to have only a minor effect on the overall results.

### Shared-evidence reasoning increases panel-based robustness of correctness across models

We next evaluated whether shared-evidence retrieval-augmented reasoning improves the panel-based robustness of correctness across model variability. Panel-based robustness of correctness was defined, for each question, as the fraction of models in the evaluated 34-model panel producing the correct answer, capturing sensitivity to model choice within this panel rather than average single-model performance (Figure 4; Table S5). Across all 169 questions, shared-evidence reasoning produced a clear upward shift in robustness. Mean robustness increased from 0.74 under zero-shot inference to 0.81 under shared-evidence reasoning, and the median increased from 0.79 to 0.94. The proportion of questions in the high-robustness bin rose from 50% to 72%, while the medium-robustness fraction declined from 41% to 17%, and the low-robustness fraction increased from 9% to 11%. The paired per-question change was statistically significant (*p* = 5.6 × 10^−9^; rank-biserial r = 0.45), with a median ΔR of +0.07, corresponding to approximately one additional model answering correctly per question (Table S6). These results indicate that shared-evidence reasoning increased panel-based reproducibility of correct decisions in this model panel.

**Figure 4:**
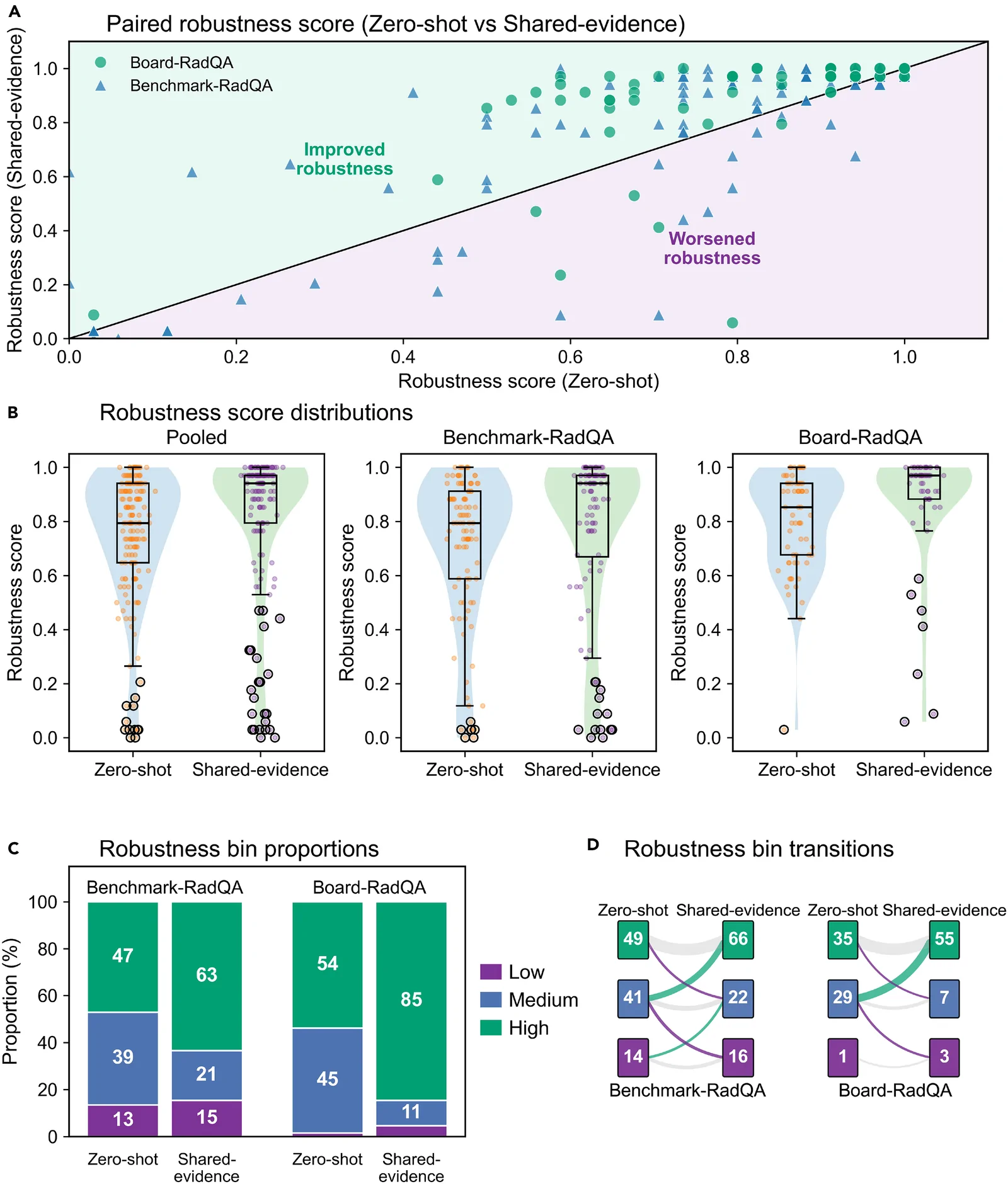
Panel-based robustness of correctness across models under zero-shot and shared-evidence inference Panel-based robustness (R) was defined per question as the fraction of the 34-model panel selecting the reference-standard answer. (A) Per-question robustness under zero-shot (*x* axis) vs. shared-evidence inference (*y* axis), shown separately for Benchmark-RadQA and Board-RadQA. The identity line indicates no change. Points above the line reflect improved robustness under shared-evidence inference, whereas points below indicate decreased robustness. (B) Violin plots with embedded boxplots showing the distribution of robustness scores under zero-shot and shared-evidence inference for pooled questions and for each dataset separately. Points represent per-question values; boxplots indicate median and interquartile range. (C) Proportion of questions falling into low, medium, and high robustness bins under zero-shot and shared-evidence inference for each dataset. (D) Transition diagrams illustrating per-question shifts between robustness categories from zero-shot to shared-evidence inference in Benchmark-RadQA and Board-RadQA. Flows depict movements between bins, highlighting dominant medium-to-high transitions as well as rare downward shifts.

Subset stratification confirmed the same pattern for the second primary endpoint. In the non-converted Board-RadQA subset (*n* = 65), shared-evidence reasoning significantly increased panel-based robustness (mean ΔR = +0.08; *p* = 7.4 × 10^−6^; r = 0.56), with the high-robustness fraction rising from 54% to 85%. A consistent effect was observed in the Benchmark-RadQA subset (*n* = 104), where mean panel-based robustness increased by +0.06 (*p* = 1.2 × 10^−4^; r = 0.38), with the high-robustness fraction rising from 47% to 63%. These results confirm that the primary robustness finding was preserved in the non-converted subset and was not driven solely by the conversion-affected Benchmark-RadQA items (Note S2).

### Robustness gains coexist with rare large collapse cases

Although panel-based robustness most often improved or remained stable under shared-evidence reasoning, a small subset of questions exhibited pronounced decreases (Table S5). Across all 169 questions, panel-based robustness improved in 45 (27%), remained unchanged in 112 (66%), and decreased in 12 (7%) (Table S6). Some decreases were large in magnitude, including collapse events with ΔR = −0.74, reflecting coordinated shifts in correctness across many models rather than isolated failures. While upward transitions dominated overall, these tail events demonstrate that shared-evidence reasoning can synchronize incorrect decisions in rare cases. Thus, shared-evidence inference improves panel-based robustness substantially more often than it degrades it, yet rare robustness collapses persist and represent safety-relevant failure modes.

### Output verbosity is a weak and inconsistent proxy for correctness

We next examined, as an exploratory analysis, whether response length relates to correctness within each inference condition and dataset (Table S7; Figure S1). All comparisons were performed within inference method to isolate verbosity-correctness associations from systematic length differences introduced by the shared-evidence pipeline. Across all 169 questions, verbosity showed only a minimal association with correctness under zero-shot inference and no meaningful association under shared-evidence inference. Under zero-shot inference, the median verbosity was slightly higher for correct responses than for incorrect responses, 280 vs. 256 tokens, with a significant but negligible effect size (*p* = 0.020; Cliff’s δ = 0.04). Although significant, the effect size was negligible, indicating only a very small difference in response length between correct and incorrect outputs. Under shared-evidence inference, median verbosity was nearly identical between correct and incorrect responses, 660 vs. 668 tokens (*p* = 0.833; Cliff’s δ = −0.004), with a negligible effect size and no evidence of a relationship between response length and correctness.

### Consensus strength and robust correctness are only partially coupled

We examined, as a secondary analysis, whether stronger inter-model consensus corresponds to more robust correctness across models by relating majority fraction to panel-based robustness at the per-question level (Table 4; Figure 5). Across all 169 questions, consensus strength and panel-based robustness were coupled under both zero-shot inference (ρ = 0.88, *p* = 1.8 × 10^−55^) and shared-evidence reasoning (ρ = 0.87, *p* = 6.8 × 10^−54^).

**Table 4:** Relationship between inter-model consensus strength and correctness under zero-shot and shared-evidence reasoning

Metric	Pooled dataset		Board-RadQA dataset		Benchmark-RadQA dataset	
	Zero-shot	Shared-evidence	Zero-shot	Shared-evidence	Zero-shot	Shared-evidence
**Majority agreement strength stratified by correctness**						

Correct [n]	153	149	61	59	92	90
Incorrect [n]	16	20	4	6	12	14
Median M (Correct)	0.88	0.97	0.91	0.97	0.85	0.96
Median M (Incorrect)	0.56	0.59	0.15	0.21	0.58	0.67
Mean M (Correct)	0.85	0.91	0.88	0.95	0.82	0.89
Mean M (Incorrect)	0.48	0.53	0.15	0.27	0.50	0.68
*p* value (Mann-Whitney U)	6.1 × 10^−8^	1.0 × 10^−10^	8.4 × 10^−4^	2.8 × 10^−5^	6.8 × 10^−5^	3.3 × 10^−6^
Cliff’s δ	0.82	0.87	1.00	0.99	0.71	0.77
Effect size	Large	Large	Large	Large	Large	Large

**Rank-based association between consensus strength and robustness**						

Questions [n]	169	169	65	65	104	104
Spearman ρ	0.88	0.87	0.81	0.69	0.93	0.96
*p* value (Spearman test)	1.8 × 10^−55^	6.8 × 10^−54^	2.3 × 10^−16^	1.6 × 10^−10^	1.4 × 10^−45^	4.0 × 10^−56^

This table summarizes how inter-model consensus strength relates to correctness and robust correctness across zero-shot and shared-evidence inference, reported for pooled questions and dataset-specific subsets (Board-RadQA and Benchmark-RadQA). In the upper section, consensus strength is quantified for each question as the majority fraction (M), defined as the proportion of models selecting the modal answer option among the 34-model panel. Questions are stratified according to whether the majority decision matches the reference-standard answer (“Correct”) or not (“Incorrect”). Median and mean majority fractions are reported for each stratum. Statistical comparisons between correct-majority and incorrect-majority questions are performed within each method and dataset using a two-sided Mann-Whitney U test, with Cliff’s delta (δ) reported as an effect size. All *p* values in the upper section correspond to these unpaired rank-based comparisons. In the lower section, the coupling between consensus strength and robust correctness is quantified using Spearman’s rank correlation coefficient (ρ), computed across questions for each inference method and dataset. Robust correctness is defined as the fraction of models selecting the ground-truth answer. Statistical significance of the monotonic association is assessed using two-sided Spearman correlation tests. All *p* values in the lower section correspond to tests of the null hypothesis ρ = 0.

**Figure 5:**
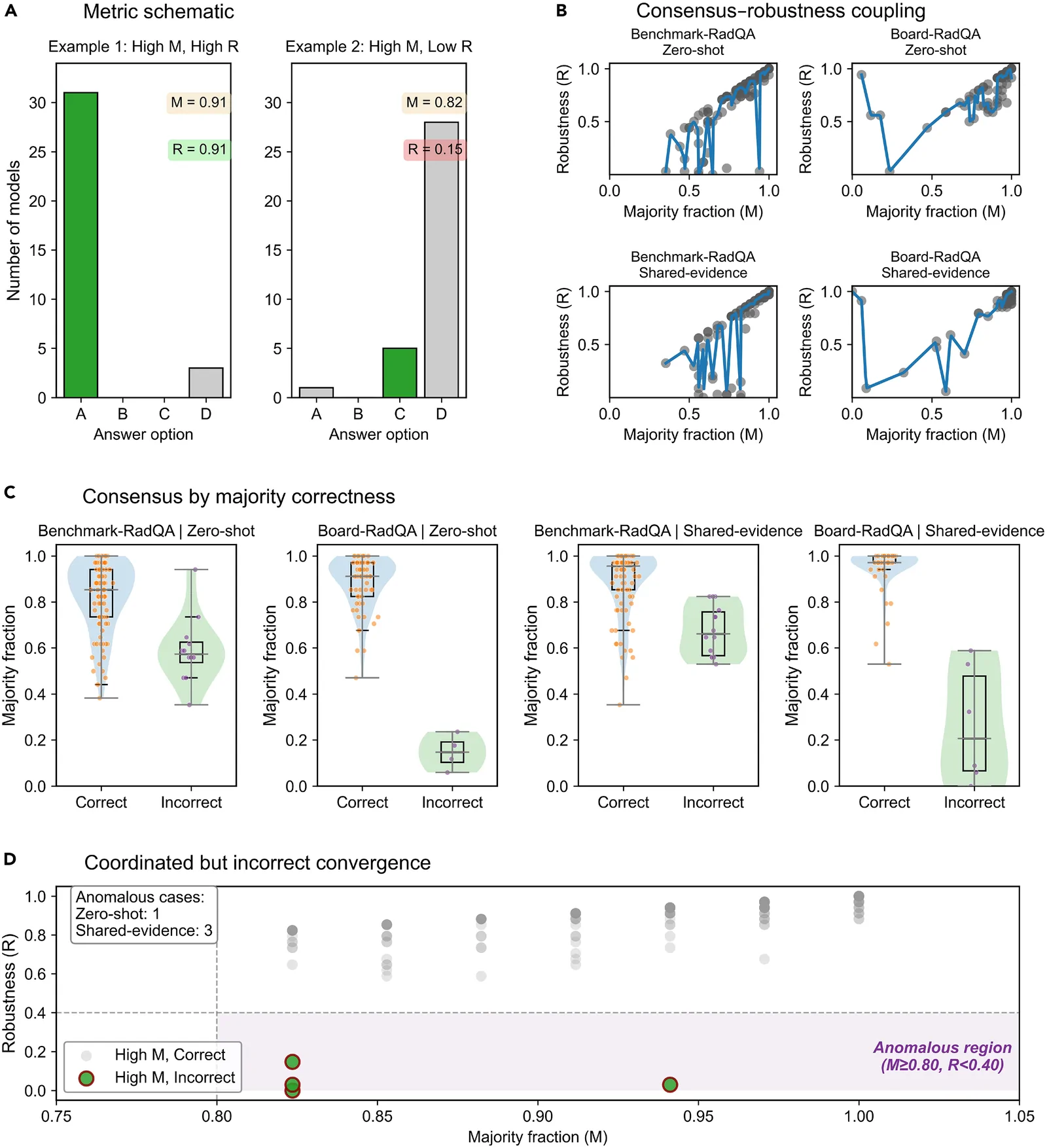
Coupling between consensus strength and robust correctness and coordinated incorrect convergence Consensus strength was quantified as the majority fraction (M) and robust correctness as the fraction of models selecting the ground-truth answer (R), both computed per question. Results are shown separately for Benchmark-RadQA and Board-RadQA under zero-shot and shared-evidence inference. (A) Metric schematic illustrating representative scenarios. Example 1 shows high consensus and high robustness (high M, high R), where most models agree and most are correct. Example 2 shows high consensus but low robustness (high M, low R), where many models agree on the same answer but few select the correct option. (B) Scatterplots of robustness (R) vs. majority fraction (M) at the per-question level for each dataset and inference condition. Each point represents one question. Lines illustrate monotonic trends corresponding to Spearman rank correlations, quantifying the strength of coupling between agreement and correctness. (C) Distribution of majority fractions stratified by whether the majority decision was correct or incorrect. Violin plots with embedded boxplots display per-question majority fractions for correct-majority and incorrect-majority cases within each dataset and inference strategy. (D) Identification of coordinated but incorrect convergence. Points denote per-question (M, R) pairs, with the shaded region highlighting the predefined anomalous zone (M ≥ 0.8 and R < 0.4). High-consensus incorrect cases are marked, illustrating instances in which strong inter-model agreement coexisted with low robustness of correctness.

At the majority-decision level, correct majorities exhibited higher agreement than incorrect majorities. In the pooled zero-shot analysis, the median majority fraction was 0.88 for correct vs. 0.56 for incorrect majorities (*p* = 6.1 × 10^−8^; Cliff’s δ = 0.82, large). Under shared-evidence inference, the corresponding medians were 0.97 vs. 0.59 (*p* = 1.0 × 10^−10^; Cliff’s δ = 0.87, large). However, high agreement did not guarantee correctness. We identified four cases of high-consensus, low-robustness behavior: one under zero-shot (1/169, 1%) and three under shared-evidence inference (3/169, 2%) (Table S8). Thus, consensus and panel-based robustness were strongly aligned on average under both strategies, but consensus should not be interpreted as a stand-alone marker of correctness because coordinated incorrect convergence can still occur.

### Exploratory clinical severity annotations suggest heterogeneous error consequences

To characterize the clinical risk profile of model failures, we analyzed radiologist-assigned severity labels for all incorrect answer options across all 169 questions (Figure 6). Severity annotation was performed independently by two board-certified radiologists (L.A. and T.T.N.) and one senior radiology resident (F.B.O.), all blinded to model identities, inference strategies, and collective performance metrics. For each incorrect answer option, raters classified the anticipated clinical consequence as low, moderate, or high severity (Table S9).

**Figure 6:**
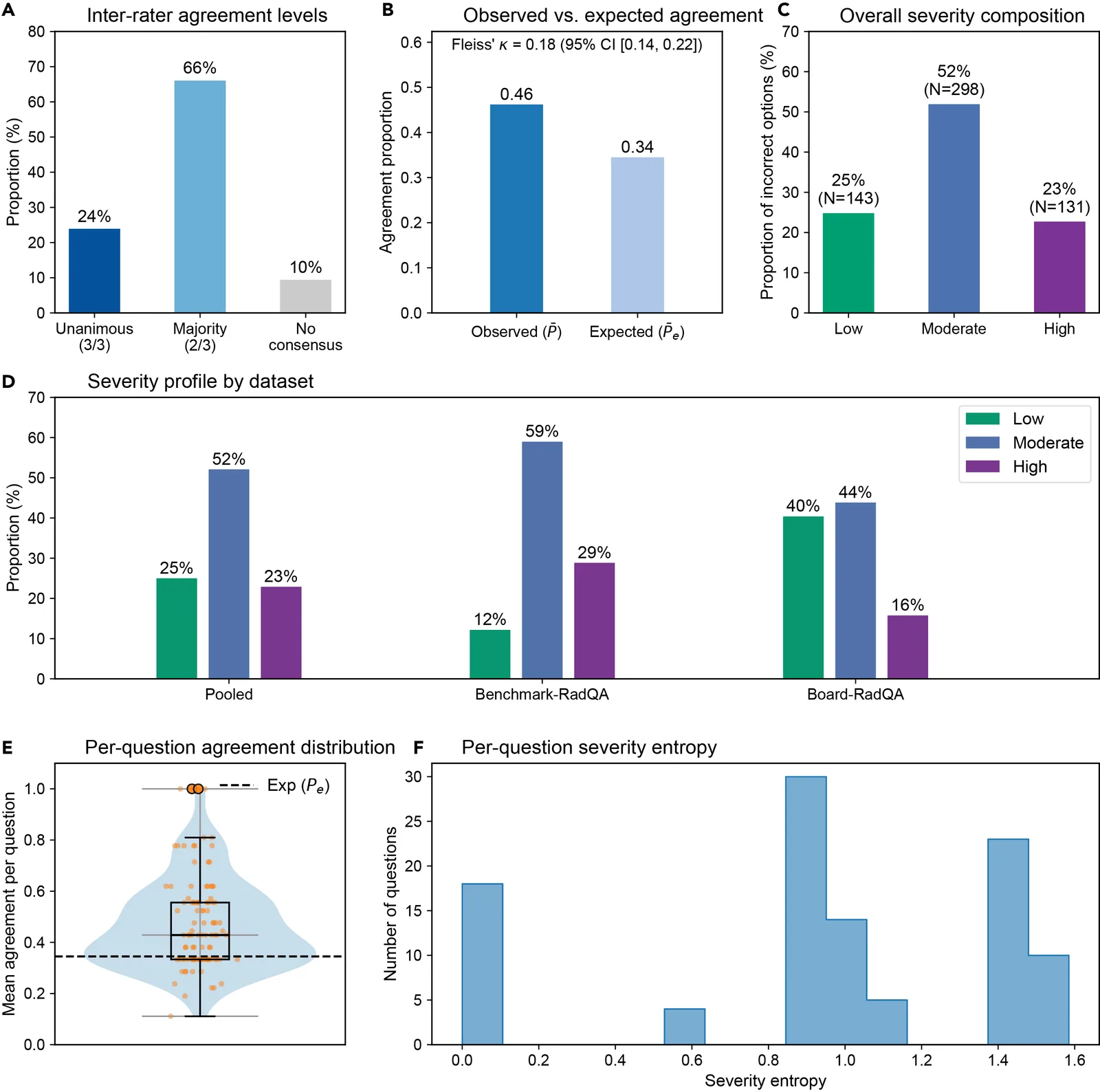
Clinical severity distribution and inter-rater agreement for incorrect answer options Clinical severity of incorrect multiple-choice options was independently assessed by three radiologists and categorized as low, moderate, or high anticipated clinical impact. (A) Inter-rater agreement levels across all annotated options. Bars show the proportion of options with unanimous agreement (3/3), majority agreement (2/3), or no consensus among raters. (B) Observed vs. expected agreement proportions used to compute Fleiss’ κ. The observed mean agreement exceeded the expected agreement under chance, yielding a κ value of 0.18. (C) Overall severity composition across all annotated incorrect options (*n* = 572). Bars show the proportion and absolute counts of low-, moderate-, and high-severity categories. (D) Severity profile stratified by dataset (pooled, Benchmark-RadQA, and Board-RadQA), displaying the percentage of incorrect options assigned to each severity level within each subset. (E) Distribution of mean per-question agreement across raters. The violin plot with embedded boxplot shows variability in agreement at the question level; the dashed horizontal line indicates the expected agreement under chance. (F) Distribution of per-question severity entropy, quantifying heterogeneity of severity labels across incorrect options within each question. Higher entropy values reflect greater dispersion of severity categories, indicating that clinical impact of errors is not concentrated in a single severity level.

In total, 572 incorrect model outputs were linked to options annotated as low, moderate, or high severity according to predefined clinical impact criteria, which are detailed in the methods section. Overall percent agreement among raters was 72%. Mean observed agreement was *Pˉ* = 0.46 and expected agreement under chance was *Pˉ*_*e*_ = 0.34, yielding a Fleiss’ κ of 0.18. This indicates minimal agreement beyond chance despite moderate raw agreement. Aggregated severity labels were not dominated by low-severity errors: low-severity errors comprised 0.25, moderate-severity errors 0.52, and high-severity errors 0.23 of cases. However, because chance-corrected inter-rater agreement was minimal, these proportions should be interpreted cautiously. The severity analysis is therefore best viewed as exploratory and hypothesis-generating. It suggests that residual errors may include clinically consequential options, but it does not provide a robust or definitive quantification of clinical risk.

### Shared-evidence reasoning reshapes collective decision structure without eliminating coordinated error

Across analyses, a consistent structural pattern emerges. For the two primary endpoints, shared-evidence retrieval-augmented reasoning reduced inter-model dispersion and increased panel-based robustness of correctness as primary findings, while majority consensus increased as a secondary finding. These effects indicate that shared structured evidence aligned heterogeneous models toward more concentrated answer distributions and more reproducible correctness within the evaluated model panel.

Secondary and exploratory analyses provided additional context. Majority consensus increased overall, but agreement amplification was not uniformly correctness-preserving, and shared-evidence reasoning sometimes concentrated models around incorrect answers. Consensus strength and panel-based robustness were strongly aligned on average under both inference strategies, yet high-consensus and low-robustness cases persisted. Strong agreement therefore did not guarantee broadly shared correctness. Verbosity did not function as a reliable signal of validity, with largely overlapping length distributions between correct and incorrect responses. Exploratory clinical severity annotations suggested that residual incorrect outputs may include clinically consequential options, but low chance-corrected inter-rater agreement limits strong conclusions from this analysis.

Single-model accuracy changes were heterogeneous across the 34 LLMs (Table 1). Several smaller and mid-sized models showed statistically significant gains under shared-evidence inference, whereas many higher-performing models changed little. Thus, population-level improvements in dispersion and panel-based robustness do not imply uniform gains for every individual model, underscoring the value of evaluating collective behavior beyond per-model accuracy alone.

## Discussion

This study evaluated how a fixed shared-evidence retrieval-augmented reasoning^8^^,^^13^ condition reshapes collective decision behavior across a heterogeneous panel of 34 LLMs on 169 expert-curated, text-only, multiple-choice radiology questions spanning two datasets.^8^^,^^24^ Rather than focusing on single-model accuracy, we analyzed reliability under model variability by decomposing collective behavior into prespecified primary endpoints, namely inter-model decision stability and panel-based robustness of correctness, and secondary or exploratory dimensions including consensus strength, consensus-robustness coupling, verbosity-correctness associations, and clinical severity annotations of incorrect answer options. The design held retrieval and evidence synthesis constant across models by providing identical structured evidence reports for each question, allowing us to evaluate how different models use the same structured evidence rather than their independent retrieval, planning, or tool-use abilities.

Shared-evidence retrieval-augmented reasoning was associated with lower inter-model dispersion, reflected by a significant reduction in entropy in the pooled analysis. This pattern suggests that identical structured evidence reports tend to align heterogeneous models toward fewer distinct answer modes. Importantly, lower entropy reflects stronger coordination among models but does not imply higher validity.^25^ Majority agreement also increased, indicating more concentrated collective decisions. However, agreement amplification was not exclusively correctness-preserving. Although most increases in consensus occurred in questions with correct majorities, some questions showed increased agreement around incorrect answers. These observations reinforce that stability and consensus are structural properties of collective behavior rather than direct indicators of correctness. A three-level ablation isolating retrieval access from structured synthesis indicated that retrieval alone concentrated decisions but did not significantly improve panel-based robustness, whereas the structured synthesis step added significant gains in both decision concentration and robustness, locating the robustness improvement specifically in the evidence-synthesis stage rather than in retrieval access per se.

Panel-based robustness of correctness increased under shared-evidence retrieval-augmented reasoning, indicating that a larger fraction of models in the evaluated 34-model panel reached the correct answer for many questions. This metric captures reproducibility of correctness within the tested model panel, not an intrinsic property of the question or a deployment-invariant reliability estimate. Improvements were not uniform. A small subset of questions exhibited pronounced robustness decreases, including rare collapse events in which many models shifted away from the correct answer under shared-evidence inference. These coordinated failures illustrate that shared structured context can occasionally synchronize errors across models, reducing the protective value of model diversity.^15^^,^^18^ Thus, the shared-evidence condition modified the distribution of errors, combining broader gains in panel-based correctness with residual tail-risk behavior.

The relationship between consensus and correctness further clarifies this structure. Consensus strength and panel-based robustness were strongly correlated across questions under both inference strategies, indicating that stronger agreement generally coincided with higher fractions of correct models. Correct majority decisions also showed substantially higher agreement than incorrect majorities. Nevertheless, high agreement did not guarantee correctness. A small number of high-consensus, low-robustness cases occurred under both inference conditions, demonstrating that coordinated incorrect convergence can arise even when models appear highly aligned. Agreement therefore remains an informative but imperfect signal of reliability.^29^

Verbosity behaved similarly as a weak indicator of validity.^30^ Response lengths for correct and incorrect outputs largely overlapped, and effect sizes were negligible. Under shared-evidence inference, no meaningful relationship between verbosity and correctness was observed. These findings suggest that response length or explanatory detail should not be interpreted as a reliable proxy for correctness, particularly in pipelines that systematically increase output length through structured reasoning steps.^21^^,^^31^

Clinical severity annotations provided an exploratory safety-relevant perspective. Incorrect model outputs were often mapped to moderate or high aggregated severity categories,^32^ but inter-rater agreement^28^ beyond chance was minimal despite moderate raw agreement. This low chance-corrected agreement indicates substantial uncertainty in assigning discrete severity categories to incorrect answer options. The finding likely reflects the contextual complexity of judging downstream clinical consequences, which depend on clinical setting, management pathway, opportunity for correction, and reversibility of harm. Accordingly, the severity analysis should be interpreted as preliminary and hypothesis-generating rather than as a robust quantification of clinical risk. It suggests that residual errors may include clinically consequential options but does not establish a definitive estimate of patient harm.

The secondary analyses should be interpreted with appropriate restraint. Majority consensus, consensus-robustness coupling, verbosity-correctness associations, and clinical severity annotations provide useful descriptive views of model behavior, but they were not the primary endpoints and were not subjected to formal multiple-comparison adjustment outside the prespecified per-model analyses. We therefore treat these findings as exploratory and hypothesis-generating. Their role is to contextualize the primary findings on decision dispersion and panel-based robustness, not to establish independent evidence of clinical reliability.

Our study has limitations. First, the evaluation is based on 169 curated, text-only, multiple-choice radiology questions across two datasets. This paired,^33^ per-question design enables controlled comparison of inference conditions and precise estimation of structural metrics such as entropy, panel-based robustness, and consensus coupling. However, the total sample size limits statistical power for fine-grained subgroup analyses, including question-type stratification, pathology-specific effects, or retrieval-pattern sensitivity.^34^ Larger and more diverse datasets, including broader pathology coverage and varying difficulty levels, would allow more granular modeling of collapse-prone scenarios and stronger statistical certainty for tail-risk analyses. Relatedly, panel-based robustness depends on the composition of the evaluated model panel; because our panel intentionally combined proprietary and open-weight systems, small and very large models, and general-purpose and medically adapted variants, robustness values should be interpreted as descriptive statistics over this specific 34-model panel rather than deployment-invariant reliability estimates. Furthermore, the evaluated models are not fully independent. Several models belong to related families, including Qwen, Llama, Gemma, DeepSeek, and OpenAI variants, and may share training data, architectural design, or alignment procedures. Consensus and robustness may therefore partly reflect lineage-related correlation rather than independent agreement among unrelated systems. Because related models can produce correlated correct and incorrect answers, panel-based robustness in particular should be interpreted as a descriptive statistic over this specific panel rather than as evidence of independent agreement, and we did not attempt to derive an independence-adjusted reliability estimate from these correlated outputs. Second, the multiple-choice format abstracts away from the open-ended reasoning typical of real clinical reporting. It supports reproducible scoring across many models, but it does not capture free-text report generation, differential diagnosis formulation, or interactive clinical reasoning. Third, the evaluation is text-only and does not incorporate imaging data or multimodal clinical context. Real-world radiology workflows integrate images, prior examinations, reports, clinical history, and evolving clinical information.^1^^,^^22^ The present findings should therefore be interpreted as evidence about controlled text-based radiology QA under model variability, not as direct evidence of clinical decision-support performance or deployment readiness.^35^^,^^36^ Fourth, answer adjudication relied on structured binary correct or incorrect classification with rule-based extraction of the final explicitly stated answer option when multiple options were mentioned. Although this approach ensured consistency and reproducibility, it assumes that the final explicitly stated option reflects the model’s intended decision. In rare cases of ambiguous phrasing or complex answer formulations, this rule-based determination may imperfectly capture the model’s true intent. Fifth, the shared-evidence pipeline was fixed across all models to isolate inter-model variability under identical structured context.^33^ This design ensures controlled comparisons but also constrains architectural diversity in retrieval and report synthesis. Alternative retrieval sources, ranking strategies, prompt templates, evidence synthesis models, or report formats may produce different stability, robustness, and collapse patterns. Our three-level ablation begins to disentangle these components, attributing the robustness gains primarily to structured evidence synthesis rather than to retrieval access alone; however, it varies only the presence of synthesis, and systematic ablation of retrieval depth, evidence diversity, ranking strategy, or report structure would further clarify which design choices drive robustness gains versus synchronized failures. Sixth, all models received identical retrieved context per question. While this design isolates how different models respond to shared evidence, it may amplify correlated errors when retrieval is misleading, incomplete, omits relevant information, or overemphasizes a misleading cue.^18^ Because the structured reports were generated by a fixed GPT-4o-mini orchestration model, synthesis errors, unsupported statements, or omissions at this intermediate stage could propagate to all evaluated models and contribute to coordinated failures. Future systems could explore retrieval diversity, independent evidence-quality checks, source-grounding verification, ensemble retrieval strategies, or indicators that detect low confidence or conflicting context before it is broadcast to all models. Seventh, clinical severity labeling is inherently subjective and context dependent. Although three blinded clinicians independently annotated incorrect options, chance-corrected agreement was low, reflecting the nuanced and scenario-specific nature of harm assessment.^37^ Larger annotation panels, structured adjudication procedures, or explicitly probabilistic severity frameworks may improve reliability and transparency.^32^^,^^38^

In summary, fixed shared-evidence retrieval-augmented reasoning modified the collective structure of controlled text-based radiology QA under model variability. In this setting, it was associated with reduced inter-model dispersion and higher panel-based robustness of correctness, while majority consensus also increased as a secondary finding. At the same time, consensus remained an imperfect indicator of correctness, and coordinated incorrect convergence as well as rare robustness collapses were still observed. Improvements in collective, panel-based robustness therefore coexist with residual tail-risk behavior. These findings suggest that evaluating retrieval-augmented reasoning pipelines through average accuracy or agreement alone is insufficient. Complementary analyses of decision stability, panel-based robustness, consensus-correctness coupling, and carefully interpreted exploratory safety annotations can provide a more complete view of reliability in text-based radiology QA under model variability.

## Methods

### Ethics statement

All procedures followed relevant guidelines and regulations. This study used previously published and expert-curated question datasets and did not involve patients, identifiable personal data, or human subjects. Institutional review board approval and informed consent were therefore not required.

### Datasets

We evaluated models on 169 multiple-choice radiology questions drawn from two expert-curated datasets: a Benchmark Radiology QA dataset (Benchmark-RadQA; *n* = 104), sourced from the RadioRAG study,^24^ and a board-style Radiology QA dataset (Board-RadQA; *n* = 65), sourced from the RaR study.^8^

### Benchmark-RadQA (*n* = 104; RadioRAG derived)

Benchmark-RadQA^24^ is a benchmark-style radiology QA dataset constructed by combining two components from the RadioRAG study: RSNA-RadioQA^24^ and ExtendedQA^24^ (Note S4). RSNA-RadioQA was curated from 80 peer-reviewed cases in the Radiological Society of North America (RSNA) Case Collection. Questions were created from the clinical history and image characteristics described in the case documentation and figure captions, while images themselves were not included to enforce a text-only evaluation setting. During curation, differential diagnoses explicitly listed by original case authors were excluded to reduce leakage of the correct answer. The dataset spans 18 radiology subspecialties (with at least five questions per subspecialty in the source dataset), reflecting a broad coverage of diagnostic radiology topics (Table S1). ExtendedQA was developed to probe generalization and reduce the risk of data contamination in evaluation. It consists of 24 radiology questions developed and validated by board-certified radiologists with substantial diagnostic radiology experience (5–14 years).^24^ These questions were designed to reflect realistic clinical diagnostic scenarios that were not previously available online in the same form as standard case collections. RSNA-RadioQA is derived from a publicly accessible case collection; therefore, some degree of training data cannot be fully excluded for any evaluated model family. ExtendedQA and Board-RadQA were included to probe generalization under reduced online availability of question formulations.

Because RadioRAG-derived^24^ Benchmark-RadQA items were converted to multiple-choice format, we used the standardized preprocessing described in the RaR paper to harmonize format and scoring across all 104 Benchmark-RadQA questions. Question stems and reference-standard answers were preserved during conversion. To standardize evaluation, three clinically plausible distractors were generated for every question to produce four answer choices per item, consisting of one correct answer and three distractors. Candidate distractors were drafted using OpenAI’s GPT-4o and o3 models, but these models were used only to generate preliminary alternatives and did not determine the ground truth. All candidate distractors were subsequently reviewed by a board-certified radiologist. The review focused on clinical plausibility, diagnostic relevance, appropriate difficulty, absence of obvious implausibility, and avoidance of distractors that were misleading because of ambiguity rather than legitimate diagnostic challenge. Distractors that failed review were removed or revised before inclusion. The final answer options were therefore expert-curated and identical across all evaluated models and inference conditions. The resulting Benchmark-RadQA dataset used here contains 104 multiple-choice questions with four options per question (A–D).

### Board-RadQA (*n* = 65; board-style, RaR derived)

Board-RadQA^8^ is a set of 65 board-style radiology questions aligned with German radiology board examination domains. Questions were developed and validated by board-certified radiologists with 9–10 years of experience and reflect core diagnostic knowledge emphasized in structured radiology training. According to the RaR study,^8^ these questions and their formulations are not available in online case collections or known LLM training corpora, reducing the likelihood of training-data overlap. Board-RadQA questions were formatted as five-option multiple-choice items with a single reference-standard correct answer. The dataset is publicly available for research use as reported in the RaR study.^8^

Across both datasets, identical question text, answer options, and ground-truth labels were used across all models and inference conditions.

### Model panel

We evaluated a fixed panel of 34 heterogeneous language models spanning a wide range of parameter scales, training paradigms, and access models. The panel was designed to approximate realistic deployment heterogeneity rather than to rank individual systems. The evaluated models included proprietary and open-weight systems from multiple families. Concretely, the panel comprised the following: Claude-Sonnet-4.6, Gemini-3.1-Pro (Preview),^39^ MiniMax-M2.5,^40^ GLM-5,^41^ LFM2.5-1.2B-Thinking, Kimi-K2.5,^42^ Palmyra-X5, MiMo-V2-Flash,^43^ Ministral-8B, Mistral Large, Llama3.3-8B,^44^^,^^45^ Llama3.3-70B,^46^^,^^47^ Llama3-Med42-8B,^48^ Llama3-Med42-70B,^49^ Llama4-Scout-16E,^32^ DeepSeek R1-70B,^47^ DeepSeek-R1,^47^ DeepSeek-V3,^48^ Qwen 2.5–0.5B,^49^ Qwen 2.5-3B,^49^ Qwen 2.5-7B,^49^ Qwen 2.5–14B,^49^ Qwen 2.5–70B,^49^ Qwen 3-8B,^50^ Qwen 3-235B,^50^ GPT-3.5-turbo, GPT-4-turbo,^51^ o3, GPT-5,^52^ GPT-5.2, MedGemma-4B-it,^53^ MedGemma-27B-text-it,^53^ Gemma-3-4B-it,^54^^,^^55^ and Gemma-3-27B-it.^54^^,^^55^ These models range from sub-billion to very-large-scale architectures and include general-purpose, instruction-tuned, and medically adapted variants. Detailed specifications, access modes, and version identifiers are provided in Table S2. All models were run using the lowest-variance decoding settings available for each API (e.g., minimum supported temperature; top-p set to 1 or disabled when applicable). For OpenAI reasoning models (e.g., o3), sampling parameters such as temperature/top-p are not user-configurable, so we used the model defaults. We generated one response per question per inference condition.^8^

### Inference conditions

Each model answered every question under two inference strategies: zero-shot inference and shared-evidence retrieval-augmented reasoning (Notes S5 and S6).

In the zero-shot condition, models received only the question stem and answer options and were instructed to select the single best answer. No external retrieval, tools, or iterative reasoning scaffolds were provided. A standardized prompt template was used across models and datasets, instructing the model to act as a medical expert and to select exactly one option. The exact prompt templates are provided in Note S6.

In the shared-evidence retrieval-augmented reasoning condition, models received additional structured context generated by a fixed retrieval-augmented^13^^,^^24^ orchestration pipeline adapted from prior radiology-focused retrieval and reasoning frameworks. Retrieval and evidence synthesis were not performed independently by each evaluated model. Instead, a single fixed orchestration pipeline generated one structured evidence report per question. The pipeline comprised three sequential stages: (1) automated extraction and abstraction of key diagnostic concepts from the question stem; (2) multi-step evidence retrieval restricted to Radiopaedia.org,^23^ a peer-reviewed radiology knowledge base; and (3) synthesis of the retrieved content into a standardized structured report generated by a single fixed orchestrator model. This report was then provided unchanged to all evaluated models for the corresponding question before answer selection.

The final answer was always generated by the evaluated model itself. The orchestration engine therefore functioned only as a context-construction mechanism and not as a decision-maker. This design isolates how different models use the same external evidence rather than comparing their independent retrieval, planning, or tool-use abilities.^8^ Accordingly, the shared-evidence condition should be interpreted as one fixed retrieval-and-synthesis design, not as a general evaluation of retrieval-augmented reasoning systems. Prompt templates and representative examples of retrieved-context formatting are provided in Notes S5 and S6 to support reproducibility.

Retrieval depth and evidence selection were fixed by the orchestration pipeline. For each answer option, the pipeline performed option-specific searches using the option text and diagnostic concepts extracted from the question stem. Retrieval was restricted to Radiopaedia.org through domain-restricted search queries and post-retrieval source verification. Each option-specific research module was allowed up to four retrieval attempts, including initial focused searches and refined contextual searches when evidence was insufficient. Retrieved results were deduplicated before synthesis. Evidence selection prioritized Radiopaedia pages that directly addressed the diagnosis, imaging finding, or differential diagnosis represented by the answer option. The resulting report summarized supporting and contradicting evidence for each option where available, while the final answer was left to the evaluated model.

Because the same structured report was provided to all models, report quality represents a pipeline-level factor. The report-generation step was source-restricted to Radiopaedia and designed to summarize retrieved evidence rather than select the final answer. Nevertheless, any unsupported statement, omission, or overemphasis introduced during report synthesis could affect all downstream models. We therefore interpret coordinated failures as possible consequences of the full shared-evidence pipeline, including retrieval and synthesis, rather than attributing them solely to the final-answering models.

To disentangle the contribution of external retrieval from that of structured evidence synthesis, we additionally evaluated a standard retrieval-augmented (standard RAG) condition on a matched subset of models for which all three conditions were available (*n* = 15 models; Note S3). In this condition, each question was presented together with the top-ranked passages retrieved from the same Radiopaedia-restricted source used by the shared-evidence pipeline, without the multi-step orchestration, option-wise decomposition, or synthesized structured report. This yields a three-level comparison, zero-shot (closed-book), standard RAG (retrieved passages), and shared-evidence retrieval-augmented reasoning (synthesized structured report), in which retrieval access is introduced at the second level and structured synthesis is added at the third. All three conditions used the identical question set, answer options, and ground-truth labels and were analyzed with the same paired, per-question framework described above.

### Answer adjudication

Responses were first evaluated against the reference-standard correct option for each question using a structured adjudication procedure with binary correct or incorrect classification. For responses classified as correct, scoring was based directly on the reference-standard option. For responses classified as incorrect, the generated text was automatically reviewed using rule-based matching to identify the final explicitly stated answer option, which was treated as the model’s definitive selection. This procedure ensured a consistent and reproducible determination of the model’s intended answer, including cases in which multiple options were mentioned within a single response.^8^

### Evaluation framework and experimental design

Our evaluation targets collective behavior across models under model variability. Analyses were defined at the per-question level and paired across inference conditions. For each question *q*, each of the *N* = 34 models produced one response under zero-shot and one under shared-evidence inference, yielding 68 responses per question and 11,492 total responses across 169 questions. Unless otherwise stated, the question is the statistical unit of analysis, and comparisons are paired^34^ by question. The model panel contains several related model families, and model outputs should not be interpreted as statistically independent samples. Majority fraction and panel-based robustness are therefore used as descriptive measures of collective behavior within the evaluated panel, rather than as formal ensemble estimates that require independence among models. Correlated outputs may arise from shared training data, architectures, alignment procedures, or provider-specific design choices.

### Inter-model decision stability

Inter-model decision stability was used to quantify how consistently a heterogeneous panel of language models converged on the same answer when presented with an identical radiology question under a fixed inference strategy. The underlying rationale is that, when multiple independent models are exposed to the same task, the dispersion of their discrete decisions provides a measure of collective stability that is distinct from correctness.

For each radiology question and each inference condition, we collected one final answer from each of the 34 models. Answers were restricted to the predefined multiple-choice options of the question, yielding a finite categorical outcome space. For a given question under a given method, the distribution of model decisions over the available options defines an empirical categorical distribution. If *n*(*o*) denotes the number of models selecting option *o* and *N* = 34 denotes the total number of models, the empirical probability of option *o* is defined as $p\left(o\right)=\frac{n\left(o\right)}{N}$.

Decision stability was quantified using Shannon entropy,^25^ defined as:

(Equation 1) $$H=-\sum_{o\in O}p\left(o\right)\log\;p\left(o\right),$$

where *O* denotes the set of available answer options for that question. By convention, terms with *p*(*o*) = 0 contribute zero to the sum. Entropy equals zero when all models select the same option and increases as responses become more evenly distributed across options. In this framework, entropy captures dispersion of decisions without reference to their correctness. A single entropy value was computed for each question under each inference condition. This produced paired entropy measurements per question, one for zero-shot inference and one for shared-evidence retrieval-augmented reasoning. Inter-model stability was therefore operationalized as a question-level property that could be directly compared across inference conditions while holding the question constant.

To characterize how the inference strategy influences collective decision structure, paired differences in entropy between conditions were computed at the question level. These paired values form the basis for distributional summaries. Importantly, this stability metric is correctness-agnostic and is intended to capture coordination structure rather than validity.^15^^,^^16^ This separation allows subsequent analyses to disentangle agreement, correctness, and robustness as distinct dimensions of model behavior.

### Majority decision behavior

Majority decision behavior was analyzed to characterize how strongly a panel of heterogeneous models converges on a single answer and whether such convergence aligns with the reference standard.^56^ Whereas entropy captures the full dispersion of responses, the majority-based analysis focuses on the dominant collective decision and the strength with which it is supported.^25^

For each radiology question under each inference condition, we considered the set of final answer options selected by the 34 models. Let *n*(*o*) denote the number of models selecting option *o* from the option set *O*. The majority option *o*∗ is defined as the option with the highest frequency among model responses, *o*∗ = *argmax*_*o*∈*O*_*n*(*o*). The strength of consensus was quantified by the majority fraction, $M=\frac{max_{o\in O}n\left(o\right)}{N}$, where *N* = 34 is the number of models. This quantity represents the proportion of models supporting the most common answer and lies between the reciprocal of the number of options and 1. Higher values indicate stronger concentration of decisions on a single option. Because several models belong to related families, majority fraction should be interpreted as a descriptive measure of answer concentration across the evaluated panel, not as independent evidence accumulation across unrelated systems.

To relate consensus to validity, the majority decision was compared with the ground-truth answer for that question. Majority correctness was treated as a binary property, indicating whether the majority option coincided with the reference standard. This labeling does not alter the definition of consensus itself but allows subsequent analyses to examine how agreement and correctness interact. Each question yielded two majority fractions, one under zero-shot inference and one under shared-evidence retrieval-augmented reasoning. The question-level change in consensus strength was defined as the paired difference Δ*M* = *M*_*sharedevidence*_−*M*_*zero*-*shot*_. This paired formulation ensures that differences are evaluated within the same question, controlling for variation in topic and difficulty.^34^ For interpretive analyses, questions were grouped according to whether consensus strength increased, decreased, or remained unchanged between conditions and whether the majority decision under the shared-evidence condition was correct or incorrect. These categorizations serve to describe how shifts in collective agreement relate to decision validity without assuming that consensus is itself a reliability metric.

### Panel-based robustness of correctness across models

Panel-based robustness of correctness was evaluated to quantify how consistently a question was answered correctly within the evaluated 34-model panel and to what extent correctness depended on model choice within this panel.^18^ Unlike accuracy, which is defined per model as the fraction of questions answered correctly, panel-based robustness is defined per question as the fraction of evaluated models that answer correctly, capturing cross-model reproducibility of correct decisions within the chosen panel.^11^^,^^16^ This metric should not be interpreted as an intrinsic property of the question, the retrieval-augmented reasoning pipeline, or a deployment-invariant reliability estimate, because its value depends on the composition of the model panel. It may also be affected by lineage correlations among related models, which can increase similarity in both correct and incorrect responses. For each question under each inference condition, the correctness of each model response was determined relative to the reference standard. Let *c*_*i*_∈{0,1} denote the correctness indicator for model *i*, where *c*_*i*_ = 1 indicates a correct answer and *c*_*i*_ = 0 otherwise. With *N* = 34 models, robustness of correctness for a given question and method is defined as:

(Equation 2) $$R=\frac{1}{N}\sum_{i=1}^{N}c_{i}\text{,}$$

which corresponds to the empirical probability that a randomly selected model from the evaluated 34-model panel answers the question correctly. This quantity lies in the unit interval, with higher values indicating that correctness is reproducible across many independent model instances rather than driven by a small subset.

To facilitate interpretable summaries of reliability regimes, robustness values were additionally stratified into three ordinal categories representing low, intermediate, and high cross-model consistency. These categories were defined by fixed thresholds on *R* and used exclusively for descriptive transition analyses, whereas all statistical testing was conducted on the continuous robustness values to avoid discretization artifacts.^57^ Each question yielded two robustness values, one under zero-shot inference and one under shared-evidence retrieval-augmented reasoning. The question-level change in robustness was defined as the paired difference Δ*R* = *R*_*sharedevidence*_−*R*_*zero*-*shot*_, which isolates the effect of the inference strategy while holding the question constant.^33^ Positive values indicate that a larger fraction of models reached the correct answer under the shared-evidence condition, whereas negative values indicate reduced cross-model consistency of correctness.

For descriptive transition analyses, questions were further characterized according to whether their robustness category increased, decreased, or remained stable between inference conditions. This transition view is intended to reveal structural shifts in reliability regimes, such as movement from fragile to consistently correct behavior or, conversely, coordinated degradations. Importantly, panel-based robustness is interpreted as a descriptive measure of cross-model stability of correctness within the evaluated model panel, not as a proxy for clinical validity, task difficulty, or deployment-invariant reliability.

### Output verbosity as a confidence proxy

To examine whether commonly exposed verbosity signals relate to decision validity, we analyzed the association between response length and correctness at the level of individual model outputs. The underlying question is whether longer or more detailed responses systematically correspond to higher likelihood of correctness, which would make verbosity a plausible proxy for confidence.^19^^,^^20^ Each model response produced under each inference condition constituted one observation. For every response, correctness was determined relative to the reference standard, yielding a binary indicator *c*∈{0,1}. Two quantitative verbosity measures were extracted from the textual outputs. Reasoning length was defined as the number of tokens in the model’s explanatory reasoning segment when present and summary length as the number of tokens in the final answer or summary segment.^58^ Token counts were computed using the OpenAI’s tiktoken tokenizer, a fast byte-pair encoding with the *cl*100*k*_*base* encoding. Formally, for a given verbosity measure *L* and inference method *m*, we consider the conditional distributions *L*∣*c* = 1,*m* and *L*∣*c* = 0,*m*. These represent the length distributions for correct and incorrect responses, respectively, under the same inference condition. The analysis tests whether these distributions differ in location or spread, without assuming any specific parametric form.

All comparisons were performed within inference condition, so that zero-shot responses were only compared to other zero-shot responses and shared-evidence responses only to shared-evidence responses. This isolates the relationship between verbosity and correctness from systematic length differences induced by the inference pipeline itself. Throughout, verbosity measures are treated strictly as descriptive behavioral signals. They are not interpreted as calibrated confidence scores,^17^ and no assumption is made that longer outputs imply greater reliability or safety.^19^^,^^20^

### Coupling between consensus strength and robust correctness

To examine whether stronger inter-model agreement corresponds to more reliable correctness across models, we analyzed the coupling between consensus strength and robustness at the per-question level.^18^^,^^59^ This analysis integrates two previously defined quantities: the majority fraction, which captures how concentrated model answers are on a single option, and the robustness score, which captures how many models independently arrive at the correct answer. The goal is to determine whether these two dimensions track each other or can diverge. For each question *q* and inference method *m*, consensus strength was represented by the majority fraction *M*_*q*,*m*_, defined as the proportion of models selecting the most frequent answer option, and robust correctness was represented by the robustness score *R*_*q*,*m*_, defined as the fraction of models that selected the ground-truth option. Both quantities lie in the unit interval [0, 1] but capture different aspects of collective behavior: *M*_*q*,*m*_ is agnostic to correctness, whereas *R*_*q*,*m*_ is correctness-anchored.

The association between consensus and robustness was quantified separately for each inference method using Spearman rank correlation coefficient.^60^ Spearman correlation was chosen because it evaluates monotonic association without assuming linearity or normality and is appropriate for bounded, non-Gaussian variables. Formally, for each method *m*, the correlation *ρ*_*m*_ was computed over paired observations (*R*_*q*,*m*_) across questions. This analysis assesses whether questions with stronger consensus also tend to exhibit higher fractions of correct models.

To further test whether higher consensus preferentially occurs on correct collective decisions, we compared the distribution of majority fractions between questions where the majority decision matched the reference standard and those where it did not. This comparison isolates whether consensus strength itself is systematically aligned with correctness at the majority-vote level rather than at the individual-model level.

Finally, we explicitly characterized consensus-related failure modes by identifying questions for which strong agreement coexisted with weak correctness. These were defined *a priori* as questions satisfying

(Equation 3) $$M_{q,m}\geq 0.8\;and\;R_{q,m}<0.4\text{,}$$

meaning that a large majority of models agreed on an answer while fewer than 40% of models selected the correct one. Such cases represent coordinated but incorrect convergence. Their frequency was summarized descriptively for each inference method. This analysis is intended to reveal structural patterns in collective behavior rather than to define safety thresholds or calibration properties.

### Clinical severity assessment of incorrect decisions

To evaluate the potential clinical risk associated with incorrect model decisions, we conducted an independent expert severity assessment performed by two board-certified radiologists (L.A. and T.T.N.) and one final-year radiology resident (F.B.O.), with 10, 8, and 5 years of clinical experience in diagnostic and interventional radiology across subspecialties. This component was designed to quantify the clinical consequence of incorrect answers rather than to reassess model accuracy. The unit of annotation was the incorrect answer option. For each question in the combined dataset of 169 radiology questions, all incorrect multiple-choice options were evaluated. Radiologists were fully blinded to model outputs, model identities, inference strategies, and all aggregate statistics derived from model behavior, including agreement levels, entropy, consensus measures, and robustness scores. They were also not informed how frequently any option had been selected by models. This blinding ensured that severity assessments reflected independent clinical judgment rather than perceptions of model performance or consensus.^61^

Radiologists were instructed to judge the likely clinical consequence if a clinician were to select a given incorrect option as the final diagnostic decision. Severity was defined in terms of potential impact on patient management and outcomes.^62^ A high-severity error corresponded to an incorrect diagnosis that could plausibly lead to substantial patient harm, major diagnostic delay, or inappropriate management with meaningful clinical risk. A moderate-severity error corresponded to an incorrect diagnosis that could lead to diagnostic delay or suboptimal management with limited or reversible clinical impact. A low-severity error corresponded to an incorrect diagnosis unlikely to meaningfully affect patient outcomes or management.^33^ Correct options were not assigned severity labels and served only to determine which options were eligible for annotation.

Inter-rater reliability across the three radiologists was quantified using percent agreement and Fleiss’ κ, which extends chance-corrected agreement measures to multiple raters.^63^ Fleiss’ κ was computed as $\kappa =\frac{Pˉ-P{ˉ}_{e}}{1-P{ˉ}_{e}}$, where *Pˉ* denotes the observed mean agreement across raters, and *Pˉ*_*e*_ denotes the expected agreement under chance. This metric was selected because severity labels are categorical and independently assigned by more than two raters.

For downstream analyses, option-level severity labels were aggregated using majority agreement across the three radiologists. In cases without a strict majority, the median severity on the ordinal scale from low to high was assigned according to a prespecified rule. These aggregated severity labels were subsequently linked to model-level failure patterns, including incorrect majority decisions, high-consensus incorrect decisions, and robustness-collapse cases.

Severity labels were used exclusively for descriptive and stratified analyses of safety-relevant failure modes. They were not incorporated into correctness scoring and did not influence entropy, consensus, or robustness calculations. In this way, clinical severity was treated as an independent safety-relevant dimension that complements, but does not redefine, statistical performance metrics. This design allows a clear separation between the frequency of errors, the consistency of errors across models, and the potential clinical consequence of those errors.^9^

### Statistical analysis

M.F., J.S., S.N., and S.T.A. performed the evaluations and statistical analyses between January and March 2026. Statistical analyses and data visualization were conducted using Microsoft Excel (Microsoft Excel for Microsoft 365 MSO [v.2602 Build 16.0.19725.20126] 64-bit) and Python (Python v.3.11.7 | packaged by Anaconda, Inc.). Specifically, the primary calculations for inter-model decision stability (entropy), majority fraction, and clinical severity reported in the text and tables were performed using Microsoft Excel. To ensure accuracy and reproducibility, these calculations were independently cross-validated. For the remaining analyses, the following packages were used: NumPy (v.1.24.0) and Pandas (v.2.0.0) for data handling, SciPy (v.1.10.0) for statistical analysis, Matplotlib (v.3.7.0) and Seaborn (v.0.12.0) for visualization, Plotly (v.5.0.0) with Kaleido (v.0.2.1) for figure export, OpenPyXL (v.3.0.0) for Excel file handling, and TikToken (v.0.5.0) for tokenization-based measurement of response length. Single-model accuracy results (Table 1) were estimated using bootstrapping with 1,000 resamples to compute means, standard deviations, and 95% confidence intervals (CIs)^64^ across the pooled dataset of 169 questions. A strictly paired design ensured identical resamples across conditions.^65^ To evaluate statistical significance for model-level comparisons between zero-shot and shared-evidence retrieval-augmented methods, exact McNemar’s test^66^ based on the binomial distribution was applied separately to each model using paired question-level outcomes. *p* values were adjusted for multiple comparisons using the false discovery rate,^67^ with a significance threshold of 0.05.

All other hypothesis tests were two-sided with a significance threshold of 0.05, and no formal adjustment for multiple comparisons was applied, and results should be interpreted in the context of multiple hypothesis testing. The primary unit of analysis was the question. Core comparisons between inference strategies used a paired^33^ per-question design, pairing zero-shot and shared-evidence results for the same question. Because several metrics are bounded and exhibited heterogeneous distributions, we emphasize distributional summaries and effect sizes alongside *p* values. To mitigate type I error inflation across multiple endpoints, we prespecified a hierarchy of outcomes. The primary outcomes were (1) inter-model decision stability, quantified by entropy, and (2) panel-based robustness of correctness. Secondary outcomes included majority fraction, consensus-robustness coupling, verbosity-correctness associations, and clinical severity distributions. Secondary analyses are interpreted as exploratory and hypothesis-generating.

For inter-model decision stability (entropy *H*), majority fraction (*M*), and robustness of correctness (*R*), we assessed systematic differences between shared-evidence and zero-shot inference using the Wilcoxon signed-rank test^68^ applied to paired per-question values. This non-parametric test was chosen because it does not assume normality and is appropriate for bounded or skewed distributions. For each metric *X*∈{*H*,*M*,*R*}, we defined the paired difference at the question level as Δ*X*_*q*_ = *X*_*q*,*sharedevidence*_−*X*_*q*,*zero*-*shot*_.

For the three-level ablation, the same paired Wilcoxon signed-rank framework was applied to each adjacent and baseline contrast (standard RAG vs. zero-shot, shared-evidence vs. zero-shot, and shared-evidence vs. standard RAG) on the matched 15-model subset, with paired differences defined as the later minus the earlier condition.

We report medians, IQRs, and distributional characteristics for both raw values and paired differences. In accordance with standard signed-rank procedures, questions with exactly zero paired difference were excluded from the Wilcoxon test statistic; the number of non-zero pairs is reported where relevant. Effect sizes for paired tests are reported as rank-biserial correlations (*r*).^69^

For comparisons between independent groups within the same inference method, we used the Mann-Whitney U test.^70^ This was applied when comparing verbosity-related proxies (reasoning length and summary length) between correct and incorrect responses and when comparing majority-fraction values between majority-correct and majority-incorrect questions. For these analyses, we report group medians, IQRs, two-sided *p* values, and effect sizes as Cliff’s delta^71^ (or rank-biserial correlation^69^ where applicable). These effect sizes provide interpretable non-parametric measures of stochastic dominance without assuming specific distributional forms.^72^^,^^73^

To quantify the association between consensus strength and robustness of correctness, we computed Spearman rank correlations between majority fraction (*M*) and robustness (*R*) separately for each inference method. Spearman correlation was selected because it captures monotonic relationships without assuming linearity or normality.^60^^,^^63^

For categorical outcomes and predefined thresholds, including majority-behavior categories, robustness-bin transitions, and predefined high-consensus (*M* ≥ 0.8) and low-robustness (*R* < 0.4) cases, we report counts and proportions relative to the relevant denominator (dataset or method). These summaries are descriptive and are not subjected to additional hypothesis testing unless explicitly stated.^74^

For radiologist severity annotations of incorrect answer options, inter-rater reliability^28^ was summarized using percent agreement and Fleiss’ κ.^64^ Severity labels are analyzed descriptively and subsequently linked to model-level failure subsets, such as incorrect-majority decisions or high-consensus failures.^10^^,^^22^ These labels are not used to modify correctness scoring or any core quantitative metric.^9^

## Resource availability

### Lead contact

Requests for further information and resources should be directed to and will be fulfilled by the lead contact, Soroosh Tayebi Arasteh (soroosh.arasteh@rwth-aachen.de).

### Materials availability

No new materials were generated in this study.

### Data and code availability

• The Benchmark-RadQA dataset (comprising RSNA-RadioQA and ExtendedQA items) is available through the original RadioRAG publication and its associated open resources.
• The Board-RadQA dataset is publicly available for research use and can be accessed as reported in the RaR study and its supplementary materials.
• The full evaluation and analysis pipeline used to compute stability, consensus, robustness, and related metrics is available at https://zenodo.org/records/21001886.^75^
• Shared-evidence retrieval-augmented inference in this study used a previously described orchestration pipeline. To support transparency and reproducibility, the implementation used for generating retrieval-augmented reports is publicly available at https://github.com/sopajeta/RaR.
• In the present study, this pipeline was treated as a fixed inference component and was not modified beyond configuration for dataset inputs. Its availability allows independent reproduction of the shared-evidence condition. The implementation relies on widely used open-source frameworks, including the following:
  oLangChain Open Deep Research: https://github.com/langchain-ai/deep-research
  oLangChain (v.0.3.25): https://github.com/langchain-ai/langchain
  oLangGraph (v.0.4.1): https://github.com/langchain-ai/langgraph
  oOpenAI Python SDK (v.1.77.0): https://platform.openai.com
  oSearXNG metasearch engine: https://github.com/searxng/searxng
  oDocker (v.25.0.2): https://www.docker.com
• Locally hosted models were run between July 1 and August 22, 2025. These models were served using vLLM v.0.9.0 (https://github.com/vllm-project/vllm). Tensor parallelism matched the number of GPUs per node; models under 3B parameters were served without tensor parallelism. The following open-weight models were evaluated, with sources listed for reproducibility:
  oQwen 2.5-0.5B: https://huggingface.co/Qwen/Qwen2.5-0.5B
  oQwen 2.5-3B: https://huggingface.co/Qwen/Qwen2.5-3B
  oQwen 2.5-7B: https://huggingface.co/Qwen/Qwen2.5-7B
  oQwen 2.5-14B: https://huggingface.co/Qwen/Qwen2.5-14B
  oQwen 2.5-70B: https://huggingface.co/Qwen/Qwen2.5-72B
  oQwen 3-8B: https://huggingface.co/Qwen/Qwen3-8B
  oQwen 3-235B: https://huggingface.co/Qwen/Qwen3-235B-A22B
  oLlama 3.3-8B: https://huggingface.co/meta-llama/Meta-Llama-3-8B
  oLlama 3.3-70B: https://huggingface.co/meta-llama/Llama-3.3-70B-Instruct
  oLlama 3-Med42-70B: https://huggingface.co/m42-health/Llama3-Med42-70B
  oLlama 3-Med42-8B: https://huggingface.co/m42-health/Llama3-Med42-8B
  oLlama4 Scout 16E: https://huggingface.co/meta-llama/Llama-4-Scout-17B-16E
  oMistral Large: https://huggingface.co/mistralai/Mistral-Large-Instruct-2407
  oMinistral 8B: https://huggingface.co/mistralai/Ministral-8B-Instruct-2410
  oGemma-3-4B-it: https://huggingface.co/google/gemma-3-4b-it
  oGemma-3-27B-it: https://huggingface.co/google/gemma-3-27b-it
  oMedgemma-4B-it: https://huggingface.co/google/medgemma-4b-it
  oMedgemma-27B-text-it: https://huggingface.co/google/medgemma-27b-text-it
  oDeepSeek-V3: https://huggingface.co/deepseek-ai/DeepSeek-V3
  oDeepSeek-R1: https://huggingface.co/deepseek-ai/DeepSeek-R1
  oDeepSeek-R1-70B: https://huggingface.co/deepseek-ai/DeepSeek-R1-Distill-Llama-70B
• OpenAI proprietary models were accessed via official API. The versions used were as follows:
  oGPT-5.2 (2025-08-07)
  oGPT-5 (2025-08-07)
  oO3 (2025-04-16)
  oGPT-4-Turbo (2024-04-09)
  oGPT-3.5-Turbo (2024-01-25)
• The OpenRouter unified API was used to access more recent models:
  ogoogle/gemini-3.1-pro-preview
  oanthropic/claude-sonnet-4.6
  oz-ai/glm-5
  oLiquidAI/LFM2.5-1.2B-Thinking
  ominimax/minimax-m2.5
  omoonshotai/kimi-k2.5
  owriter/palmyra-x5
  oxiaomi/mimo-v2-flash
• Any additional information required to reanalyze the data reported in this paper is available from the lead contact upon request.

## Acknowledgments

S.T.A. is supported by the Excellence Strategy of the German Federal Government, the Länder, and the RWTH ERS (START_526-26). F.B.O. received funding from the Clinician Scientist Program of RWTH Aachen University. S.W. is supported by BayernKI and the Deutsche Forschungsgemeinschaft (DFG) – 440719683. K.B. received grants from the 10.13039/501100000780European Union (101079894), 10.13039/100019627Bayern Innovativ, 10.13039/501100002347German Federal Ministry of Education and Research, 10.13039/100015330Max Kade Foundation, and 10.13039/100008672Wilhelm Sander Foundation. S.N. was supported by grants from the DFG (NE 2136/3-1, LI3893/6-1, and TR 1700/7-1). D.T. was supported by grants from the DFG (NE 2136/3-1, LI3893/6-1, and TR 1700/7-1) and is supported by the German Federal Ministry of Education (TRANSFORM LIVER, 031L0312A; SWAG, 01KD2215B) and the European Union’s Horizon Europe and Innovation Programme (ODELIA [Open Consortium for Decentralized Medical Artificial Intelligence], 101057091).

## Author contributions

The formal analysis and study conceptualization were conducted by M.F., J.S., and S.T.A. The original draft was written by S.T.A., S.A., M.F., and J.S. and edited by S.T.A. and S.N. The code was developed by M.F. and J.S. All the experiments were performed by M.F. and J.S., except for clinical severity assessment, which was performed by L.A., T.-T.N., and F.B.O. M.L., L.A., K.B., D.T., and S.T.A. contributed to data collection. S.W. contributed to LLM-serving infrastructure for model inference. The illustrations were designed by M.F., J.S., and S.T.A. The statistical analyses were performed by S.T.A., S.N., M.F., and J.S. L.A., F.B.O., T.-T.N., K.B., S.N., and D.T. provided clinical expertise. J.S., S.A., M.L., S.W., K.B., D.T., and S.T.A. provided clinical expertise. The study was defined by S.T.A. All authors read the manuscript and agreed to the submission of this paper.

## Declaration of interests

J.S. is partially employed at Siemens Healthineers, Germany. M.L. is employed by Generali Deutschland Services GmbH, Germany, and is on the editorial board for *European Radiology Experimental*. L.A. is a trainee on the editorial board for *Radiology: Artificial Intelligence*. S.W. was partially employed by DATEV eG, Germany. K.B. is a trainee on the editorial board for *Radiology: Artificial Intelligence*. D.T. received honoraria for lectures by Bayer, GE, Roche, AstraZeneca, and Philips and holds shares in StratifAI GmbH, Germany, and in Synagen GmbH, Germany. S.T.A. is on the editorial board for *Communications Medicine* and for *European Radiology Experimental* and is a trainee on the editorial board for *Radiology: Artificial Intelligence*.

## Declaration of generative AI and AI-assisted technologies in the writing process

During the preparation of this work, the authors used GPT-5.5 and Claude Opus 4.8 for language polishing. After using this tool, the authors reviewed and edited the content as needed and take full responsibility for the content of the publication.

## Supplemental information

Document S1. Figure S1, Tables S1–S9, and Notes S1–S6

Document S2. Article plus supplemental information
