# Supplementary material for: Retrieval-augmented reasoning reshapes collective reliability in text-based radiology question answering
Source: Patterns (N Y). 2026 Aug 19;7(10):101639. doi: 10.1016/j.patter.2026.101639 (PMC13674304; doi:10.1016/j.patter.2026.101639)
Supplement: Supplementary file 1 [file mmc1.pdf]

Patterns, Volume *7*

Supplemental information

Retrieval-augmented reasoning reshapes collective reliability in text-based radiology question answering

Mina Farajiamiri, Jeta Sopa, Saba Afza, Lisa Adams, Felix Barajas Ordonez, Tri-Thien Nguyen, Mahshad Lotfinia, Sebastian Wind, Keno Bressem, Sven Nebelung, Daniel Truhn, and Soroosh Tayebi Arasteh

Supplemental information

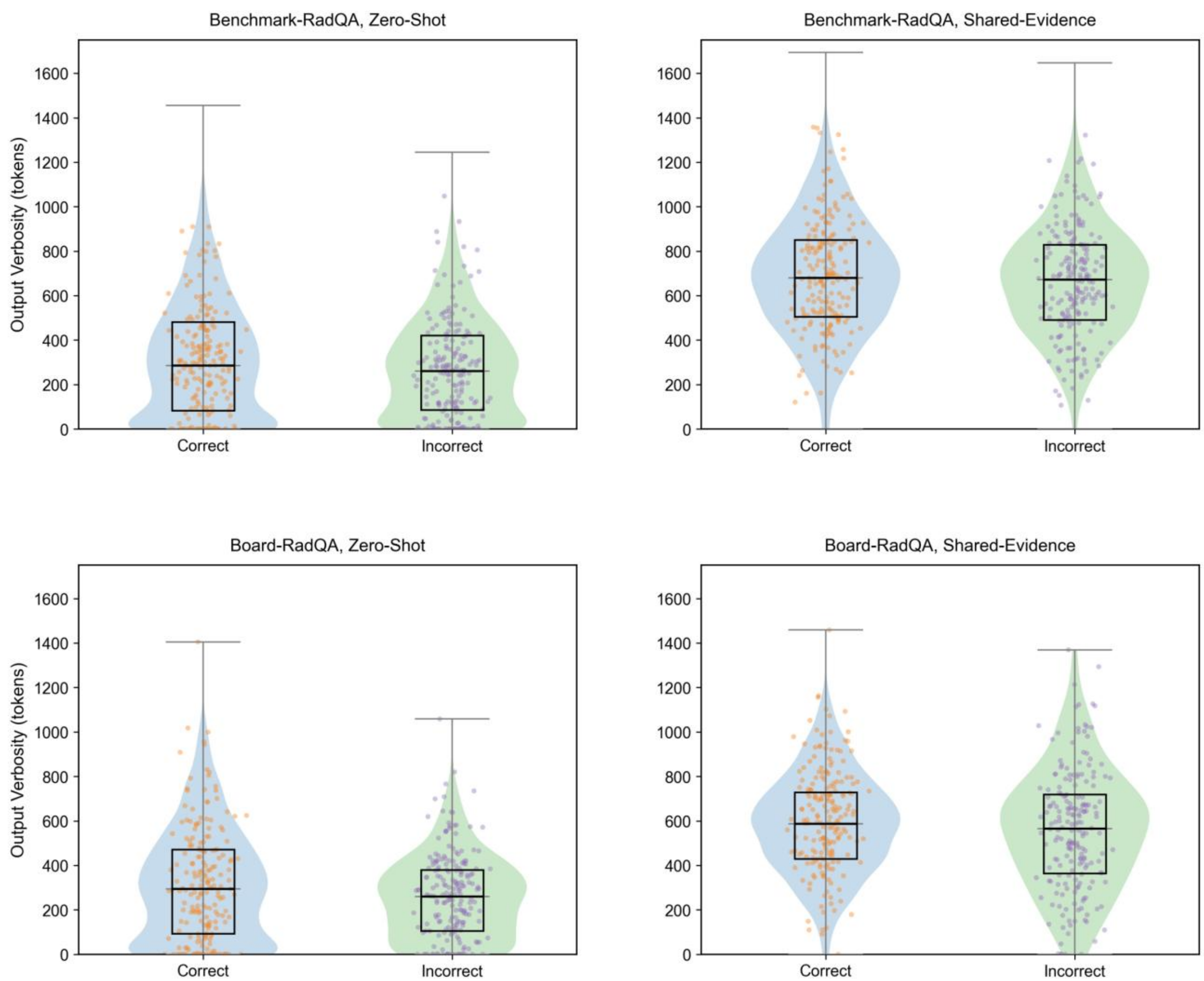

Figure S1: Output verbosity by correctness under zero-shot and shared-evidence inference. Output verbosity was quantified as total token count per model response. Distributions are shown separately by dataset and inference strategy. Violin plots with embedded boxplots display the distribution of token counts for correct and incorrect responses within each panel. Points represent individual model outputs; boxplots indicate median and interquartile range.

Table S1: Overview of datasets used to evaluate inter-model decision stability and robustness. Both datasets consist of expert-curated radiology questions spanning multiple subspecialties. Detailed dataset construction procedures are described in the original publications.

Property	Board-RadQA	Benchmark-RadQA
Questions [n]	65	104
Question type	Board-style conceptual and clinical	Case-based clinical scenarios
Authorship	Board-certified radiologists	Expert-curated, peer-reviewed sources
Subspecialty coverage	Multi-subspecialty curriculum-aligned	18 radiology subspecialties
Answer format	5-option multiple-choice	4-option multiple-choice
Images included	No	No
Primary use in prior work	Shared-evidence reasoning evaluation	Online RAG evaluation
Original source	RaR study	RadioRAG study

Table S2: Specifications of the large language models (LLMs) evaluated in this study. This table summarizes the 34 LLMs assessed under zero-shot prompting and a standard shared-evidence retrieval-augmented generation framework. For each model, we report the parameter count (in billions), category (e.g., instruction-tuned (IT), mixture of experts (MoE), reasoning-optimized, open-weights or proprietary), release date, developer, and maximum context length (in thousands of tokens). All evaluations were conducted between July 1, 2025, and February 25, 2026.

Model name	Parameters (billions)	Category	Release date	Developer	Context length (thousand tokens)
Claude-Sonnet-4.6	Undisclosed	IT, reasoning, proprietary	February 2026	Anthropic	1,000
Gemini-3.1-Pro	Undisclosed	Reasoning, proprietary	February 2026	Google DeepMind	1,000
MiniMax-M2.5	230	MoE, reasoning, open-weights	February 2026	MiniMax	197
GLM-5	744	MoE, open-source	February 2026	Z.ai	200
LFM2.5-1.2B-Thinking	1.17	IT, open-weights	January 2026	Liquid AI	32
Kimi-K2.5	1000	MoE, open-source	January 2026	Moonshot AI	256
MiMo-V2-Flash	309	MoE, open-source	December 2025	Xiaomi	256
Palmyra-X5	Undisclosed	MoE, proprietary	April 2025	Writer	1,000
Llama4-Scout-16E	17	IT, open-weights	April 2025	Meta AI	10,000
Llama3.3-8B	8	IT, open-weights	April 2024	Meta AI	8
Llama3.3-70B	70	IT, open-weights	April 2024	Meta AI	128
Llama3-Med42-8B	8	IT, clinically-aligned, open-weights	June 2024	M42 Health AI Team	8
Llama3-Med42-70B	70	IT, clinically-aligned, open-weights	June 2024	M42 Health AI Team	8
DeepSeek R1-70B	70	Reasoning, open-source	January 2025	DeepSeek	128
DeepSeek-R1	671	Reasoning, open-source	January 2025	DeepSeek	128
DeepSeek-V3	671	MoE, open-source	December 2024	DeepSeek	128
GPT-5	Undisclosed	IT, reasoning, proprietary	August 2025	OpenAI	128
GPT-5.2	Undisclosed	IT, reasoning, proprietary	December 2025	OpenAI	400
o3	Undisclosed	Reasoning, proprietary	April 2025	OpenAI	200
GPT-3.5-turbo	Undisclosed	IT, proprietary	January 2025	OpenAI	16
GPT-4-turbo	Undisclosed	IT, proprietary	April 2024	OpenAI	128
Mistral Large	123	IT, open-source	July 2024	Mistral AI	128
Ministral-8B	8	IT, open-source	October 2024	Mistral AI	128
MedGemma-4B-it	4	Gemma-3-based, multimodal, IT, clinical reasoning, open-weights	July 2025	Google DeepMind	128
MedGemma-27B-text-it	27	Gemma-3-based, text only, IT, clinical reasoning, open-weights	July 2025	Google DeepMind	≥ 128
Gemma-3-4B-it	4	IT, open-weights	March 2025	Google DeepMind	128
Gemma-3-27B-it	27	IT, open-weights	March 2025	Google DeepMind	128
Qwen 3-8B	8	Reasoning, MoE, open-source	April 2025	Alibaba Cloud	32
Qwen 3-235B	235	Reasoning, MoE, open-source	April 2025	Alibaba Cloud	32
Qwen 2.5-0.5B	0.5	IT, open-source	September 2024	Alibaba Cloud	32
Qwen 2.5-3B	3	IT, open-source	September 2024	Alibaba Cloud	32
Qwen 2.5-7B	7	IT, open-source	September 2024	Alibaba Cloud	131
Qwen 2.5-14B	14	IT, open-source	September 2024	Alibaba Cloud	131
Qwen 2.5-70B	70	IT, open-source	September 2024	Alibaba Cloud	131

Table S3: Per-question inter-model decision entropy under zero-shot and shared-evidence inference. This table reports Shannon entropy values, for the first 10 radiology questions of each dataset, under zero-shot and shared-evidence retrieval-augmented inference. Entropy (H) was computed from the distribution of answer choices across the 34-model panel, with higher values indicating greater dispersion of model decisions and lower values indicating stronger concentration of answers. For each question, entropy under zero-shot and shared-evidence inference is provided, along with the paired entropy change (ΔH), defined as shared-evidence minus zero-shot entropy. Negative ΔH values indicate reduced dispersion and increased inter-model stability under shared-evidence inference, whereas positive ΔH values indicate increased dispersion. Questions are grouped by dataset.

Dataset | question ID	H		ΔH
	Zero-shot	Shared-evidence	
**Benchmark-RadQA dataset**			
Benchmark-RadQA | 1	1.08	0.94	-0.14
Benchmark-RadQA | 2	1.05	0.13	-0.92
Benchmark-RadQA | 3	0.13	0.00	-0.13
Benchmark-RadQA | 4	0.00	0.13	0.13
Benchmark-RadQA | 5	1.02	0.90	-0.11
Benchmark-RadQA | 6	0.93	0.95	0.02
Benchmark-RadQA | 7	0.63	0.13	-0.50
Benchmark-RadQA | 8	0.71	0.00	-0.71
Benchmark-RadQA | 9	0.49	0.35	-0.14
Benchmark-RadQA | 10	0.52	0.22	-0.29
**Board-RadQA dataset**			
Board-RadQA |1	0.00	0.00	0.00
Board-RadQA |2	0.72	0.13	-0.58
Board-RadQA |3	0.40	0.30	-0.10
Board-RadQA | 4	0.26	0.00	-0.26
Board-RadQA | 5	0.00	0.00	0.00
Board-RadQA | 6	0.00	0.00	0.00
Board-RadQA | 7	0.00	0.00	0.00
Board-RadQA | 8	0.35	0.13	-0.22
Board-RadQA | 9	1.13	0.35	-0.77
Board-RadQA | 10	0.35	0.00	-0.35

Table S4: Per-question majority agreement strength, correctness, and paired agreement changes under zero-shot and shared-evidence inference. This table reports, for the first 10 radiology questions of each dataset, the majority fraction (M) under zero-shot and shared-evidence retrieval-augmented inference, where M is defined as the proportion of the 34 models selecting the modal answer option. The paired change in agreement strength (ΔM) is calculated as shared-evidence minus zero-shot majority fraction. Positive ΔM values indicate increased inter-model agreement under shared-evidence inference, whereas negative values indicate decreased agreement. For each question, majority correctness (C) is additionally reported for both inference strategies, coded as 1 if the majority answer matches the reference standard and 0 otherwise. Questions are categorized according to whether agreement increased with a correct majority, increased with an incorrect majority, decreased, or remained unchanged. The absolute rank of non-zero ΔM values (used in the paired Wilcoxon signed-rank test) is provided to document the contribution of each question to the rank-based inference. Questions are grouped by dataset.

Dataset | question ID	M		ΔM	C		Category	Absolute rank of |ΔM|
	Zero-shot	Shared-evidence		Zero-shot	Shared-evidence		
**Benchmark-RadQA dataset**							
Benchmark-RadQA | 1	0.56	0.65	0.09	0	0	Agreement ↑ & incorrect	100.5
Benchmark-RadQA | 2	0.62	0.97	0.35	1	1	Agreement ↑ & correct	166.5
Benchmark-RadQA | 3	0.97	1.00	0.03	1	1	Agreement ↑ & correct	53.0
Benchmark-RadQA | 4	1.00	0.97	-0.03	1	1	Agreement ↓	53.0
Benchmark-RadQA | 5	0.50	0.65	0.15	1	0	Agreement ↑ & incorrect	126.0
Benchmark-RadQA | 6	0.62	0.59	-0.03	1	1	Agreement ↓	53.0
Benchmark-RadQA | 7	0.76	0.97	0.21	1	1	Agreement ↑ & correct	149.0
Benchmark-RadQA | 8	0.79	1.00	0.21	1	1	Agreement ↑ & correct	149.0
Benchmark-RadQA | 9	0.85	0.91	0.06	1	1	Agreement ↑ & correct	82.0
Benchmark-RadQA | 10	0.85	0.94	0.09	1	1	Agreement ↑ & correct	100.5
**Board-RadQA dataset**							
Board-RadQA |1	1.00	1.00	0.00	1	1	No change	0.0
Board-RadQA |2	0.79	0.97	0.18	1	1	Agreement ↑ & correct	140.0
Board-RadQA |3	0.91	0.91	0.00	1	1	No change	0.0
Board-RadQA | 4	0.94	1.00	0.06	1	1	Agreement ↑ & correct	82.0
Board-RadQA | 5	1.00	1.00	0.00	1	1	No change	0.0
Board-RadQA | 6	1.00	1.00	0.00	1	1	No change	0.0
Board-RadQA | 7	1.00	1.00	0.00	1	1	No change	0.0
Board-RadQA | 8	0.91	0.97	0.06	1	1	Agreement ↑ & correct	82.0
Board-RadQA | 9	0.12	0.06	-0.06	0	0	Agreement ↓	72.5
Board-RadQA | 10	0.91	1.00	0.09	1	1	Agreement ↑ & correct	100.5

Table S5: Per-question robustness of correctness under zero-shot and shared-evidence inference and robustness transition patterns. This table reports, for the first 10 radiology questions of each dataset, robustness of correctness under zero-shot and shared-evidence retrieval-augmented inference. Robustness is defined as the fraction of the 34 models selecting the ground-truth answer. For each inference method, the total number of correct model outputs (out of 34), the corresponding robustness score (proportion correct), and the assigned robustness bin (low, moderate, high) are provided. The paired change in robustness (ΔR) is computed as shared-evidence minus zero-shot robustness score. The transition column summarizes categorical shifts between robustness bins (for example, low → high, high → low, moderate → high, or no change), enabling identification of upward transitions, stability, and robustness collapse cases. Questions are grouped by dataset.

Dataset | question ID	Zero-shot			Shared-evidence			Delta robustness	Transition
	Total correct (out of 34) [n]	Robustness score	Robustness bin	Total correct (out of 34) [n]	Robustness score	Robustness bin		
**Benchmark-RadQA dataset**								
Benchmark-RadQA | 1	0	0.00	Low	7	0.21	Low	0.21	No Change
Benchmark-RadQA | 2	20	0.59	Medium	33	0.97	High	0.38	Improved
Benchmark-RadQA | 3	33	0.97	High	33	0.97	High	0.00	No Change
Benchmark-RadQA | 4	34	1.00	High	33	0.97	High	-0.03	No Change
Benchmark-RadQA | 5	15	0.44	Medium	6	0.18	Low	-0.26	Decreased
Benchmark-RadQA | 6	16	0.47	Medium	11	0.32	Low	-0.15	Decreased
Benchmark-RadQA | 7	22	0.65	Medium	32	0.94	High	0.29	Improved
Benchmark-RadQA | 8	26	0.76	Medium	34	1.00	High	0.24	Improved
Benchmark-RadQA | 9	29	0.85	High	31	0.91	High	0.06	No Change
Benchmark-RadQA | 10	29	0.85	High	32	0.94	High	0.09	No Change
**Board-RadQA dataset**								
Board-RadQA | 1	31	0.91	High	34	1.00	High	0.09	No Change
Board-RadQA | 2	22	0.65	Medium	33	0.97	High	0.32	Improved
Board-RadQA | 3	31	0.91	High	31	0.91	High	0.00	No Change
Board-RadQA | 4	31	0.91	High	34	1.00	High	0.09	No Change
Board-RadQA | 5	34	1.00	High	34	1.00	High	0.00	No Change
Board-RadQA | 6	34	1.00	High	34	1.00	High	0.00	No Change
Board-RadQA | 7	34	1.00	High	34	1.00	High	0.00	No Change
Board-RadQA | 8	24	0.71	Medium	33	0.97	High	0.26	Improved
Board-RadQA | 9	19	0.56	Medium	31	0.91	High	0.35	Improved
Board-RadQA | 10	31	0.91	High	34	1.00	High	0.09	No Change

Table S6: Distribution of per-question robustness of correctness across a 34-model panel for the Benchmark-RadQA and Board-RadQA datasets. Robustness is defined as the fraction of models answering a question correctly. Questions are categorized into low ($R < 0.4$), medium ($0.4 \leq R < 0.8$), and high ($R \geq 0.8$) robustness. Values represent the percentage of questions in each category or transition type. Mean Δ robustness denotes the average paired change in robustness between shared-evidence and zero-shot inference. P-values derive from paired Wilcoxon signed-rank tests on continuous robustness scores.

Metric		Pooled dataset	Benchmark-RadQA dataset	Board-RadQA dataset
Number of questions		169	104	65
High robustness	Zero-shot	50%	47%	54%
	Shared-evidence	72%	63%	85%
Medium robustness	Zero-shot	41%	39%	45%
	Shared-evidence	17%	21%	11%
Low robustness	Zero-shot	9%	14%	2%
	Shared-evidence	11%	16%	5%
Robustness increased		27%	23%	32%
Robustness decreased		7%	9%	5%
Robustness unchanged		66%	68%	63%
Mean Δ robustness		0.07	0.06	0.08
Median Δ robustness		0.06	0.03	0.06
P-value		5.6×10^{-9}	1.2×10^{-4}	7.4×10^{-6}
Effect size (rank-biserial r)		0.45	0.37	0.56

Table S7: Association between output verbosity and correctness across inference methods and datasets. This table summarizes response length (token count) distributions for correct and incorrect model outputs under zero-shot and shared-evidence retrieval-augmented inference, stratified by dataset. Verbosity is reported as the median number of tokens with interquartile range (IQR) for correct and incorrect responses separately, along with the median difference (correct minus incorrect). All comparisons were performed within inference method to isolate verbosity-correctness relationships from systematic length differences induced by the inference pipeline. Statistical significance was assessed using two-sided Mann-Whitney U tests comparing correct vs. incorrect responses within each condition, with Cliff's δ reported as a non-parametric effect size. Counts reflect the total number of individual model responses analyzed per condition.

Dataset	Pooled dataset		Benchmark-RadQA dataset		Board-RadQA dataset	
	Zero-shot	Shared-evidence	Zero-shot	Shared-evidence	Zero-shot	Shared-evidence
Correct [n]	4269	4664	2505	2713	1764	1951
Incorrect [n]	1477	1082	1031	823	446	259
Median verbosity (Correct)	280	660	282	702	279	596
Median verbosity (Incorrect)	256	668	259	689	250.5	593
IQR (Correct)	334	404	338	405	324	382
IQR (Incorrect)	295.0	375.8	318.0	353.0	265.0	441.5
Median difference	24.0	8.0	23.0	13.0	28.5	3.0
P-value	0.020	0.833	0.489	0.129	0.001	0.392
Cliff's δ	0.04	-0.004	0.02	0.04	0.11	0.03

Table S8: High-consensus, low-robustness failure cases. This table enumerates questions exhibiting coordinated but incorrect convergence under zero-shot or shared-evidence inference. Cases were defined a priori as questions satisfying both a high inter-model consensus strength and low robust correctness, specifically a majority fraction $M_{q,m} \geq 0.8$ and a robustness score $R_{q,m} < 0.4$, where the majority fraction denotes the proportion of models selecting the modal answer option and the robustness score denotes the fraction of models selecting the reference-standard correct answer. These cases represent scenarios in which a large majority of models converged on the same answer despite most models being incorrect. Values are reported separately for zero-shot and shared-evidence inference conditions.

Dataset | question ID	Method	Majority fraction (M)	Robustness (R)	Majority option	Correct answer
Benchmark-RadQA | 59	shared-evidence	0.82	0.00	A	D
Benchmark-RadQA | 60	shared-evidence	0.82	0.15	D	C
Benchmark-RadQA | 65	zero-shot	0.94	0.03	A	C
Benchmark-RadQA | 65	shared-evidence	0.82	0.03	A	C

Table S9: Per-question clinical severity distribution and inter-rater agreement for incorrect answer options. This table reports, for the first 10 radiology questions of each dataset, the distribution of radiologist-assigned clinical severity labels for incorrect answer options and the corresponding inter-rater agreement statistics. For every question, all incorrect answer options selected by at least one model were evaluated independently by three clinicians and categorized as low, moderate, or high severity according to predefined clinical impact criteria. For each question the number of model outputs that were incorrect, the counts of incorrect model outputs linked to answer options assigned to each severity category after aggregation, the fraction of incorrect outputs within that question falling into each severity class are given. Inter-rater agreement across the three annotators is summarized per question using the observed agreement, expected agreement under chance, and Fleiss' κ. Fleiss' κ values close to zero indicate minimal agreement beyond chance. Questions are grouped by dataset (Benchmark-RadQA and Board-RadQA).

Dataset | question ID	Incorrect model outputs [n]	Observed agreement (P̄)	Expected agreement (P̄ₑ)	Fleiss' κ	Low severity		Moderate severity		High severity	
					[n]	Probability	[n]	Probability	[n]	Probability
**Benchmark-RadQA dataset**										
Benchmark-RadQA | 1	3	0.33	0.67	-1	1	0.33	2	0.67	0	0.00
Benchmark-RadQA | 2	3	0.33	0.67	-1	0	0.00	2	0.67	1	0.33
Benchmark-RadQA | 3	3	0.33	0.67	-1	0	0.00	2	0.67	1	0.33
Benchmark-RadQA | 4	3	0.33	0.67	-1	0	0.00	2	0.67	1	0.33
Benchmark-RadQA | 5	3	0.33	0.67	-1	0	0.00	3	1.00	0	0.00
Benchmark-RadQA | 6	3	1.00	1.00	0	1	0.33	2	0.67	0	0.00
Benchmark-RadQA | 7	3	0.33	0.67	-1	0	0.00	2	0.67	1	0.33
Benchmark-RadQA | 8	3	0.33	0.67	-1	2	0.67	0	0.00	1	0.33
Benchmark-RadQA | 9	3	0.33	0.67	-1	2	0.67	1	0.33	0	0.00
Benchmark-RadQA | 10	3	0.33	0.67	-1	0	0.00	3	1.00	0	0.00
**Benchmark-RadQA dataset**										
Board-RadQA | 1	4	0.33	0.67	-1	0	0.00	3	0.75	1	0.25
Board-RadQA | 2	4	0.00	0.33	-0.5	0	0.00	3	0.75	1	0.25
Board-RadQA | 3	4	0.33	0.67	-1	0	0.00	4	1.00	0	0.00
Board-RadQA | 4	4	0.33	0.67	-1	1	0.25	3	0.75	0	0.00
Board-RadQA | 5	4	1.00	1.00	0	0	0.00	2	0.50	2	0.50
Board-RadQA | 6	4	1.00	1.00	0	0	0.00	2	0.50	2	0.50
Board-RadQA | 7	4	0.33	0.67	-1	0	0.00	1	0.25	3	0.75
Board-RadQA | 8	4	1.00	1.00	0	4	1.00	0	0.00	0	0.00
Board-RadQA | 9	4	0.33	0.67	-1	1	0.25	3	0.75	0	0.00
Board-RadQA | 10	4	0.33	0.67	-1	4	1.00	0	0.00	0	0.00

Note S1

Dataset-based results

Shared-evidence reasoning alters inter-model decision stability

To assess whether stability shifts were consistent across datasets, we repeated the entropy analysis within Board-RadQA (n = 65) and Benchmark-RadQA (n = 104) (**Table 2, Figure 2**). In Board-RadQA, entropy was lower under shared-evidence inference, with the median decreasing from 0.35 (IQR 0.50) to 0.13 (IQR 0.13) and the mean from 0.41 to 0.20. The paired shift was significant (P = 9.6×10^{-6}); rank-biserial r = −0.94), with median ΔH = −0.13 and mean ΔH = −0.20. In Benchmark-RadQA, the median decreased from 0.53 (IQR 0.56) to 0.24 (IQR 0.57) and the mean from 0.55 to 0.38. The paired shift was also significant (P = 9.4×10^{-9}); r = −0.64), with median ΔH = −0.13 and mean ΔH = −0.18. The direction of change was predominantly stabilizing in both datasets, but heterogeneity remained. In Board-RadQA, entropy decreased for 46/65 questions (71%), increased for 8/65 (12%), and was unchanged for 11/65 (17%). In Benchmark-RadQA, entropy decreased for 69/104 (66%), increased for 23/104 (22%), and was unchanged for 12/104 (12%).

Table S3 illustrates the range of per-question behavior in concrete terms. For example, Benchmark-RadQA includes several large stabilizations (e.g., question 2: H 1.05 → 0.13, ΔH = −0.92; question 8: H 0.71 → 0.00, ΔH = −0.71), but also occasional destabilization (e.g., question 4: H 0.00 → 0.13, ΔH = +0.13) and near-null change (e.g., question 6: H 0.93 → 0.95, ΔH = +0.02). Board-RadQA shows multiple near-ceiling agreement cases with H = 0 under both methods (e.g., questions 1 and 5–7), alongside substantial stabilizations when baseline dispersion exists (e.g., question 9: H 1.13 → 0.35, ΔH = −0.77; question 2: H 0.72 → 0.13, ΔH = −0.58). These examples underscore that shared-evidence inference often compresses answer distributions, but does not impose a uniform direction of change on every question. As in the main text, these stability shifts are correctness-agnostic and should be interpreted as changes in coordination structure rather than validity**.**

Dataset-stratified consensus strength and correctness

We evaluated majority-vote agreement strength separately within Board-RadQA (n = 65) and Benchmark-RadQA (n = 104) to assess consistency across datasets (**Table 3, Table S4, Figure 3**). In both datasets, majority fractions were higher under shared-evidence inference and paired shifts were significant when restricting the Wilcoxon signed-rank test to non-zero ΔM pairs. Board-RadQA showed a median majority fraction increase from 0.91 (IQR 0.18) under zero-shot to 0.97 (IQR 0.06) under shared-evidence inference, with median ΔM = 0.03 (P = 1.5×10^{-4}). Benchmark-RadQA increased from 0.82 (IQR 0.27) to 0.94 (IQR 0.21), with a larger median ΔM = 0.06 (P = 5.7×10^{-7}). These results indicate that agreement strengthening under shared-evidence inference was present in both datasets, with a larger typical gain in Benchmark-RadQA.

The distribution of agreement-shift categories further highlights dataset differences in how consensus changes relate to correctness. In Board-RadQA, agreement increased with a correct majority in 40/65 questions (62%), increased with an incorrect majority in 1/65 (2%), decreased in 10/65 (15%), and showed no change in 14/65 (22%). In Benchmark-RadQA, agreement increased with a correct majority in 55/104 (53%), increased with an incorrect majority in 10/104 (10%), decreased in 20/104 (19%), and was unchanged in 19/104 (18%). Thus, while both datasets showed more agreement increases than decreases, Benchmark-RadQA had a higher proportion of cases where agreement increased around an incorrect majority.

Table S4 provides per-question examples that illustrate these patterns. For instance, Benchmark-RadQA includes cases where agreement increases substantially with a correct majority (e.g., question 2: M 0.62 → 0.97, ΔM = +0.35) and cases where agreement increases while the majority remains incorrect (e.g., question 1: M 0.56 → 0.65, ΔM = +0.09, majority incorrect under both methods; question 5: M 0.50 → 0.65, ΔM = +0.15, majority correctness shifts from correct to incorrect). Board-RadQA contains many ceiling or near-ceiling cases with no change (e.g., question 1: M = 1.00 in both conditions) as well as typical agreement increases with correct majorities (e.g., question 2: M 0.79 → 0.97, ΔM = +0.18). Together, these stratified results complement the pooled finding: shared-evidence inference generally strengthens consensus, but the correctness implications of stronger agreement remain question- and dataset-dependent, and agreement amplification can also occur around incorrect decisions.

Shared-evidence reasoning increases robustness of correctness across models

Dataset-stratified analyses revealed consistent but heterogeneous robustness shifts (**Tables S5** and **S6**). In the Benchmark-RadQA dataset (104 questions), mean robustness increased from 0.71 to 0.77 and the median from 0.79 to 0.94. The share of high-robustness questions rose from 47% to 63%, primarily driven by medium-to-high transitions, while low-robustness questions remained non-trivial (13% to 15%). The paired robustness shift was significant (P = 1.2×10^{-4}; r = 0.38). In the Board-RadQA dataset (65 questions), baseline robustness was higher. Mean robustness increased from 0.80 to 0.88 and the median from 0.85 to 0.97. High-robustness questions increased from 54% to 85%, with a marked reduction in the medium category (45% to 11%), while low-robustness cases remained rare but increased slightly in absolute terms. The paired effect was significant (P = 7.4×10^{-6}; r = 0.56).

Thus, both datasets showed upward robustness shifts, but the magnitude and transition structure differed.

Robustness gains coexist with rare but severe collapse cases

Dataset-level breakdown clarifies the structure of these collapse events (**Tables S5** and **S6**). In the Benchmark-RadQA dataset, 9 of 104 questions (9%) showed reduced robustness. These

included several medium-to-low or high-to-medium transitions with ΔR between −0.06 and −0.50. In the Board-RadQA dataset, 3 of 65 questions (5%) exhibited robustness decreases, including one medium-to-low collapse with ΔR = −0.74. These events reflect coordinated model shifts under shared structured retrieval. Although infrequent, their magnitude underscores that robustness gains coexist with structured tail-risk behavior.

Output verbosity is a weak and inconsistent proxy for correctness

In the Benchmark-RadQA dataset, verbosity was not associated with correctness under either inference strategy. Under zero-shot inference, median verbosity was 282 tokens for correct responses and 259 for incorrect responses (P = 0.489; Cliff's δ = 0.02). Under shared-evidence inference, median verbosity was 702 tokens for correct responses and 689 tokens for incorrect responses (P = 0.129; Cliff's δ = 0.04). Effect sizes were negligible in both cases. In the Board-RadQA dataset, zero-shot inference showed a statistically significant but small separation, with median verbosity of 279 tokens for correct responses and 250.5 tokens for incorrect responses (P = 0.001; Cliff's δ = 0.11). This difference did not persist under shared-evidence reasoning (596 vs. 593 tokens; P = 0.392; Cliff's δ = 0.03). Thus, verbosity-correctness associations were weak, dataset-dependent, and not strengthened by shared-evidence retrieval.

Consensus strength and robust correctness are only partially coupled

Dataset-stratified analyses revealed differences in coupling strength. Under zero-shot inference, correlations were strong in both datasets (Benchmark-RadQA: ρ = 0.93, P = 1.4×10^{-45}; Board-RadQA: ρ = 0.81, P = 2.3×10^{-16}). Under shared-evidence reasoning, the association strengthened further in Benchmark-RadQA (ρ = 0.96, P = 4.0×10^{-56}) but weakened in Board-RadQA (ρ = 0.69, P = 1.6×10^{-10}). Large separations between correct and incorrect majorities were observed in both datasets (all Mann-Whitney comparisons significant; Cliff's δ large). Median majority agreement was consistently higher for correct than for incorrect questions in both datasets and inference modes. These findings indicate that while pooled coupling remains strong, shared-evidence reasoning modifies the strength of consensus-robustness alignment in a dataset-dependent manner.

Note S2

Sensitivity analysis by dataset conversion status

Because the RadioRAG-derived Benchmark-RadQA items were converted to multiple-choice format before expert review, we repeated the primary analyses separately in the non-converted Board-RadQA subset and the conversion-affected Benchmark-RadQA subset. This sensitivity analysis was designed to assess whether the main findings on inter-model decision stability and panel-based robustness of correctness were preserved outside the converted Benchmark-RadQA items.

In the non-converted Board-RadQA subset (n = 65), shared-evidence inference reduced inter-model decision entropy compared with zero-shot inference. Median entropy decreased from 0.35 to 0.13, with mean entropy decreasing from 0.41 to 0.20. The paired change was statistically significant among non-zero differences (n = 49; P = 9.6×10^{-6}, z = −4.43, r = −0.94), with a median ΔH of −0.13.

In the Benchmark-RadQA subset (n = 104), shared-evidence inference likewise reduced entropy. Median entropy decreased from 0.53 to 0.24, and mean entropy decreased from 0.55 to 0.38. The paired change was again statistically significant among non-zero differences (n = 94; P = 9.4×10^{-9}, z = −5.74, r = −0.64), with a median ΔH of −0.13.

Consistent effects were observed in the pooled dataset (n = 169). Median entropy decreased from 0.48 to 0.13, with mean entropy decreasing from 0.50 to 0.40. The paired change was statistically significant among non-zero differences (n = 143; P = 5.6×10^{-9}, z = −5.83, r = −0.93), with a median ΔH of −0.13. These findings indicate that the primary entropy result was preserved in the non-converted Board-RadQA subset and was not attributable solely to conversion of Benchmark-RadQA items.

We next examined panel-based robustness of correctness. In Board-RadQA (n = 65), shared-evidence reasoning produced a pronounced upward shift in robustness. The proportion of questions in the high-robustness bin rose from 54% under zero-shot inference to 85% under shared-evidence reasoning, while the medium-robustness fraction declined from 45% to 11%. The low-robustness fraction increased from 2% to 5%. Mean panel-based robustness increased by ΔR = +0.08, and the paired per-question change was statistically significant (Wilcoxon signed-rank P = 7.4×10^{-6}; rank-biserial r = 0.56). Robustness improved for 32% of questions, decreased for 5%, and remained unchanged for the remaining questions.

In Benchmark-RadQA (n = 104), shared-evidence reasoning likewise shifted the robustness distribution upward. The high-robustness fraction increased from 47% to 63%, the medium-robustness fraction decreased from 39% to 21%, and the low-robustness fraction changed from 13% to 15%. Mean panel-based robustness increased by ΔR = +0.06, and the paired change remained statistically significant (P = 1.2×10^{-4}; r = 0.38). Robustness improved for 23% of questions, decreased for 9%, and remained unchanged for the remaining questions.

As a secondary analysis, we also examined the majority fraction. In Board-RadQA, the median majority fraction increased from 0.91 to 0.97 under shared-evidence inference (median ΔM = 0.03; n = 51 non-zero pairs; P = 1.5×10^{-4}, z = −3.80). In this subset, 40 items showed increased agreement with a correct majority decision, one item showed increased agreement with an incorrect majority decision, 10 items showed decreased agreement, and 14 showed no change. In Benchmark-RadQA, the median majority fraction increased from 0.82 to 0.94 (median ΔM = 0.06; n = 85 non-zero pairs; P = 5.7×10^{-7}, z = −5.00). In this subset, 55 items showed increased agreement with a correct majority decision, 10 showed increased agreement with an incorrect majority decision, 20 showed decreased agreement, and 19 showed no change.

Overall, both primary findings, reduced inter-model entropy and increased panel-based robustness of correctness under shared-evidence inference, were preserved in the non-converted Board-RadQA subset. This indicates that the main collective-behavior results were not driven solely by the multiple-choice conversion of Benchmark-RadQA items.

Note S3

Three-level ablation: retrieval access vs. structured evidence synthesis

To determine whether the effects of the shared-evidence pipeline are driven by external retrieval access or by structured evidence synthesis, we evaluated a standard retrieval-augmented (standard RAG) condition alongside zero-shot inference and shared-evidence retrieval-augmented reasoning. The ablation used the matched subset of 15 models evaluated under all three conditions on the identical 169-question set. In the standard RAG condition, each question was accompanied by the top-ranked passages retrieved from the same Radiopaedia-restricted source, without the multi-step orchestration, option-wise decomposition, or synthesized structured report used in the shared-evidence condition. For models for which stochastic sampling was available, the per-question model-level answer was taken as the majority-vote selected option across samples, consistent with the single-decision representation used throughout the study. All paired contrasts used two-sided Wilcoxon signed-rank tests with paired differences defined as the later minus the earlier condition; rank-biserial correlations are reported as paired effect sizes. Because the ablation uses a 15-model subset, absolute metric values differ from the 34-model panel reported in the main text and should be compared across conditions rather than against the full-panel values.

Across the pooled set, all three metrics shifted monotonically from zero-shot to standard RAG to shared-evidence reasoning. Median inter-model entropy decreased from 0.70 under zero-shot to 0.57 under standard RAG and to 0.35 under shared-evidence reasoning; median majority fraction increased from 0.87 to 0.87 to 0.93; and median panel-based robustness increased from 0.80 to 0.87 to 0.93. Introducing retrieval access alone (standard RAG vs zero-shot) significantly reduced entropy (mean $\Delta H = -0.11$; $n = 138$ non-zero pairs; $P = 0.003$; $r = -0.29$) and increased majority fraction (mean $\Delta M = +0.04$; $n = 124$; $P = 0.009$; $r = 0.27$), but did not significantly change panel-based robustness (mean $\Delta R = +0.02$; $n = 121$; $P = 0.092$; $r = 0.18$). Adding structured evidence synthesis on top of retrieval (shared-evidence vs standard RAG) produced further significant gains in all three metrics: entropy (mean $\Delta H = -0.10$; $n = 133$; $P = 0.011$; $r = -0.25$), majority fraction (mean $\Delta M = +0.03$; $n = 128$; $P = 0.003$; $r = 0.30$), and panel-based robustness (mean $\Delta R = +0.03$; $n = 126$; $P = 0.032$; $r = 0.22$). The full contrast (shared-evidence vs zero-shot) was significant for all three metrics and showed the largest effect sizes (entropy: mean $\Delta H = -0.21$; $n = 131$; $P = 6.6\times10^{-7}$; $r = -0.50$; majority fraction: mean $\Delta M = +0.07$; $n = 124$; $P = 6.7\times10^{-7}$; $r = 0.51$; panel-based robustness: mean $\Delta R = +0.05$; $n = 120$; $P = 2.9\times10^{-4}$; $r = 0.38$).

Dataset-stratified results were directionally consistent. In Benchmark-RadQA ($n = 104$), the shared-evidence vs standard RAG contrast remained significant for entropy (mean $\Delta H = -0.12$; $P = 0.015$; $r = -0.30$) and majority fraction (mean $\Delta M = +0.04$; $P = 0.009$; $r = 0.33$), with a consistent but non-significant robustness trend (mean $\Delta R = +0.03$; $P = 0.071$; $r = 0.23$); the full shared-evidence vs zero-shot contrast was significant for all three metrics (entropy $P = 3.4\times10^{-6}$; majority fraction $P = 9.5\times10^{-6}$; panel-based robustness $P = 0.004$). In the smaller Board-RadQA subset ($n = 65$), where baseline robustness was already high, the incremental shared-evidence vs standard RAG contrasts did not reach significance for any metric (entropy $P = 0.37$; majority fraction $P = 0.22$; panel-based robustness $P = 0.32$), while the full shared-evidence vs zero-shot contrast remained significant for majority fraction ($P = 0.026$; $r = 0.37$) and panel-based robustness ($P = 0.022$; $r = 0.39$).

Overall, retrieval access alone concentrated model decisions but was not sufficient to improve cross-model reproducibility of correctness, whereas the structured evidence-synthesis step contributed additional, statistically significant improvements in decision concentration and panel-based robustness beyond standard RAG. This localizes the robustness benefit of the shared-evidence pipeline to the evidence-synthesis stage rather than to retrieval access per se.

The following table lists the subset and the models in the full panel not included in the ablation.

Model name	In ablation subset	Reason not included
Llama3.3-8B	Yes	
Llama3.3-70B	Yes	
Llama4-Scout-16E	Yes	
Mistral Large	Yes	
DeepSeek-R1	Yes	
DeepSeek-V3	Yes	
Qwen2.5-0.5B	Yes	
Qwen2.5-3B	Yes	
Qwen2.5-7B	Yes	
Qwen2.5-14B	Yes	
Qwen3-8B	Yes	
Gemma-3-4B-it	Yes	
Gemma-3-27B-it	Yes	
MedGemma-4B-it	Yes	
MedGemma-27B-text-it	Yes	
Claude-Sonnet-4.6	No	Not evaluated under all three conditions
Gemini-3.1-Pro	No	Not evaluated under all three conditions
MiniMax-M2.5	No	Not evaluated under all three conditions
GLM-5	No	Not evaluated under all three conditions
LFM2.5-1.2B-Thinking	No	Not evaluated under all three conditions
Kimi-K2.5	No	Not evaluated under all three conditions
MiMo-V2-Flash	No	Not evaluated under all three conditions
Palmyra-X5	No	Not evaluated under all three conditions
GPT-3.5-turbo	No	Not evaluated under all three conditions
GPT-4-turbo	No	Not evaluated under all three conditions
GPT-5	No	Not evaluated under all three conditions
GPT-5.2	No	Not evaluated under all three conditions
o3	No	Not evaluated under all three conditions
DeepSeek R1-70B	No	Not evaluated under all three conditions
Qwen2.5-70B	No	Not evaluated under all three conditions
Llama3-Med42-8B	No	Not evaluated under all three conditions
Llama3-Med42-70B	No	Not evaluated under all three conditions
Ministral-8B	No	Variant mismatch; available checkpoint differed from main-panel version, excluded to avoid confound
Qwen3-235B	No	Variant mismatch; available variant was vision-language rather than the text-only main-panel version, excluded to avoid confound

Note S4

Datasets and question formatting

A total of 169 text-only multiple-choice radiology questions were evaluated across two expert-curated datasets.

The Benchmark-RadQA dataset contains 104 questions and is derived from the RadioRAG study[1] through combination of RSNA-RadioQA[1] and ExtendedQA[1]. RSNA-RadioQA contributes 80 questions curated from peer-reviewed cases in the Radiological Society of North America Case Collection. Questions were authored from clinical history and imaging-description text contained in the original cases, and images were intentionally excluded to enforce a text-only setting. Differential diagnoses explicitly listed by original case authors were removed during curation to reduce direct answer leakage. The source dataset spans 18 radiology subspecialties[2].

ExtendedQA contributes 24 questions that were written by a radiologist and validated by a second board-certified radiologist to probe generalization and reduce contamination risk. Because ExtendedQA was originally open-ended, it was converted to multiple-choice while preserving the original stem and reference-standard answer. To harmonize scoring across Benchmark-RadQA, each question was standardized to four options by adding three clinically plausible distractors. Distractor candidates were generated using OpenAI GPT-4o[3] and o3 as draft generators, after which review and finalization were performed by a board-certified radiologist to ensure plausibility, appropriate difficulty, and the absence of misleading or implausible options. These draft-generation models were used only to propose distractors and were not involved in determining ground truth[2].

The Board-RadQA dataset contains[2] 65 board-style questions developed and validated by board-certified radiologists to reflect domains aligned with German radiology board certification. Questions were derived from representative cases and core concepts emphasized in the Technical University of Munich radiology curriculum, with the aim of minimizing overlap with online case collections and known training corpora. Board-RadQA[2] questions were formatted as five-option multiple-choice items. Each item across both datasets has a single reference-standard correct option; Benchmark-RadQA[2] uses four options (A–D) and Board-RadQA uses five options (A–E)[2].

Note S5

Retrieval-augmented reasoning: knowledge base, tools, and orchestration workflow

Under the shared-evidence inference condition, each evaluated model is provided with a structured evidence report generated by a fixed retrieval-and-synthesis pipeline. Final answer selection is always produced by the evaluated model rather than by the orchestration engine, allowing isolation of how models use identical retrieved evidence[2].

Retrieval within shared-evidence pipeline was restricted to Radiopaedia.org, a peer-reviewed and openly accessible radiology knowledge base. Radiopaedia[4] was selected as the exclusive retrieval domain to prioritize clinically validated content and avoid heterogeneous quality across open web sources. Domain restriction was enforced by appending site-restricted filters to all queries and by performing explicit post-retrieval domain verification. Retrieval was executed through a locally hosted SearXNG meta-search engine deployed in a containerized Docker environment to support consistency and reproducibility. Retrieved results were deduplicated and formatted into standardized evidence bundles for synthesis[2]. The orchestration workflow was adapted from LangChain's Open Deep Research pipeline and implemented as a stateful directed graph using LangGraph. A supervisor component coordinated the workflow and delegated option-specific evidence gathering to research components. For each question, a neutral research plan with one section per answer option was constructed, option-specific tasks were assigned, and completed sections were synthesized into a final report containing an objective introduction and conclusion[2].

Each research module performed targeted retrieval for a single answer option. Retrieval began with focused queries using the option text and core diagnostic terms, followed by contextual queries incorporating salient clinical concepts extracted from the question stem. Retrieval was restricted to Radiopaedia.org using site-restricted queries and post-retrieval source verification. Up to four retrieval attempts were allowed per answer option. If initial results were insufficient, query refinement was performed by simplifying terms, adding synonyms, or incorporating question-specific clinical context. Retrieved results were deduplicated and organized into standardized evidence bundles. During synthesis, evidence was selected based on direct relevance to the answer option, the imaging finding, or the differential diagnosis under consideration. The structured report included option-wise evidence summaries and, when available, supporting and contradicting points. The report did not select the final answer; final answer selection was always performed by the evaluated model.

A diagnostic abstraction step guides retrieval. For each question, the Mistral Large model generates a concise comma-separated summary of key clinical concepts. This summary is used only internally for query formulation and is not shown to evaluated models. The keyword-extraction template was:

"Extract and summarize the key clinical details from the following radiology question. Provide a concise, comma-separated summary of keywords and key phrases in one sentence only. Question: {question_text}.

Summary:"

The orchestration engine responsible for planning and report writing was implemented using OpenAI GPT-4o-mini for tool use and synthesis. This orchestration model does not select the final answer. To reduce benchmark exposure during report generation, only derived keywords and the answer options (not the verbatim stem) for query formulation were provided to the orchestration model; evaluated models still received the full stem and options during evaluation. Because identical reports are provided to all evaluated models, no preferential advantage is conferred to any model family[2].

Note S6

Inference prompts, scoring, and validation procedures

Each model answered each question under two conditions: zero-shot and shared-evidence retrieval-augmented reasoning. In the zero-shot condition, only the question and options were provided. In the shared-evidence condition, the same question and options were preceded by the structured Radiopaedia[4]-derived report.

Standardized prompt templates were used.

Zero-shot template:

“You are a highly knowledgeable medical expert… {question} {options}”

Shared-evidence template:

“You are a highly knowledgeable medical expert… {report} {question} {options}”

Responses were scored against the reference-standard option. Automated option matching was applied for scalability. When multiple options were listed, correctness was assigned only when the correct option was explicitly and unambiguously selected. Manual spot checks confirmed agreement between automated scoring and human interpretation.

Constrained verification was additionally performed using Mistral Large as a binary adjudicator. The model received the response and reference answer and returned “Yes” or “No” indicating presence of the correct answer. To validate this procedure, 20 questions across five representative models (DeepSeek-R1, o3, Llama3.3-70B[5,6], MedGemma-27B-text-it[7], Qwen 2.5-3B[8]) were manually reviewed, totaling 100 responses. Complete concordance between manual and automated verification was observed[2].

Supplemental references

1. Tayebi Arasteh, S., Lotfinia, M., Bressem, K., Siepmann, R., Adams, L., Ferber, D., Kuhl, C., Kather, J.N., Nebelung, S., and Truhn, D. (2025). RadioRAG: online retrieval-augmented generation for radiology question answering. Radiol. Artif. Intell. 7, e240476. https://doi.org/10.1148/ryai.240476.
2. Wind, S., Sopa, J., Truhn, D., Lotfinia, M., Nguyen, T.T., Bressem, K., Adams, L., Rusu, M., Köstler, H., Wellein, G., Maier, A., and Tayebi Arasteh, S. (2025). Multi-step retrieval and reasoning improves radiology question answering with large language models. npj Digit. Med. 8, 790. https://doi.org/10.1038/s41746-025-02250-5.
3. OpenAI. (2023). GPT-4 technical report. Preprint at arXiv. https://doi.org/10.48550/arXiv.2303.08774.
4. Radiopaedia Australia Pty Ltd. Radiopaedia. https://radiopaedia.org.
5. Touvron, H., Lavril, T., Izacard, G., Martinet, X., Lachaux, M.-A., Lacroix, T., Rozière, B., Goyal, N., Hambro, E., Azhar, F., Rodriguez, A., Joulin, A., Grave, E., and Lample, G. (2023). LLaMA: open and efficient foundation language models. Preprint at arXiv. https://doi.org/10.48550/arXiv.2302.13971.
6. Llama Team, AI @ Meta. (2024). The Llama 3 herd of models. Preprint at arXiv. https://doi.org/10.48550/arXiv.2407.21783.
7. Sellergren, A., Kazemzadeh, S., Jaroensri, T., Kiraly, A., Traverse, M., Kohlberger, T., Xu, S., Jamil, F., Hughes, C., Lau, C., Chen, J., Mahvar, F., Yatziv, L., Chen, T., Sterling, B., Baby, S.A., Baby, S.M., Lai, J., Schmidgall, S., Yang, L., Chen, K., Bjornsson, P., Reddy, S., Brush, R., Philbrick, K., Asiedu, M., Mezerreg, I., Hu, H., Yang, H., Tiwari, R., Jansen, S., Singh, P., Liu, Y., Azizi, S., Kamath, A., Ferret, J., Pathak, S., Vieillard, N., Merhej, R., Perrin, S., Matejovicova, T., Ramé, A., Riviere, M., Rouillard, L., Mesnard, T., Cideron, G., Grill, J.-B., Ramos, S., Yvinec, E., Casbon, M., Buchatskaya, E., Alayrac, J.-B., Lepikhin, D., Feinberg, V., Borgeaud, S., Andreev, A., Hardin, C., Dadashi, R., Hussenot, L., Joulin, A., Bachem, O., Matias, Y., Chou, K., Hassidim, A., Goel, K., Farabet, C., Barral, J., Warkentin, T., Shlens, J., Fleet, D., Cotruta, V., Sanseviero, O., Martins, G., Kirk, P., Rao, A., Shetty, S., Steiner, D.F., Kirmizibayrak, C., Pilgrim, R., Golden, D., and Yang, L. (2025). MedGemma technical report. Preprint at arXiv. https://doi.org/10.48550/arXiv.2507.05201.
8. Bai, J., Bai, S., Chu, Y., Cui, Z., Dang, K., Deng, X., Fan, Y., Ge, W., Han, Y., Huang, F., Hui, B., Ji, L., Li, M., Lin, J., Lin, R., Liu, D., Liu, G., Lu, C., Lu, K., Ma, J., Men, R., Ren, X., Ren, X., Tan, C., Tan, S., Tu, J., Wang, P., Wang, S., Wang, W., Wu, S., Xu, B., Xu, J., Yang, A., Yang, H., Yang, J., Yang, S., Yao, Y., Yu, B., Yuan, H., Yuan, Z., Zhang, J., Zhang, X., Zhang, Y., Zhang, Z., Zhou, C., Zhou, J., Zhou, X., and Zhu, T. (2023). Qwen technical report. Preprint at arXiv. https://doi.org/10.48550/arXiv.2309.16609.
